# Knockout of immunotherapy prognostic marker genes eliminates the effect of the anti-PD-1 treatment

**DOI:** 10.1038/s41698-021-00175-2

**Published:** 2021-05-07

**Authors:** Naixue Yang, Fansen Ji, Liqing Cheng, Jingzhe Lu, Xiaofeng Sun, Xin Lin, Xun Lan

**Affiliations:** 1 Department of Basic Medical Science, School of Medicine, Tsinghua University, Beijing, China; 2 Peking-Tsinghua-NIBS Joint Graduate Program, Tsinghua University, Beijing, China; 3 Tsinghua-Peking Center for Life Sciences, Tsinghua University, Beijing, China; 4 Institute for Immunology, School of Medicine, Tsinghua University, Beijing, China

**Keywords:** Cancer immunotherapy, High-throughput screening

## Abstract

The efficacy of immunotherapy is largely patient-specific due to heterogeneity in tumors. Combining statistic power from a variety of immunotherapies across cancer types, we found four biological pathways significantly correlated with patient survival following immunotherapy. The expression of immunotherapy prognostic marker genes (IPMGs) in these pathways can predict the patient survival with high accuracy not only in the TCGA cohort (89.36%) but also in two other independent cohorts (80.91%), highlighting that the activity of the IPMGs can reflect the sensitivity of the tumor immune microenvironment (TIME) to immunotherapies. Using mouse models, we show that knockout of one of the IPMGs, *MALT1*, which is critical for the T-cell receptor signaling, can eliminate the antitumor effect of anti-PD-1 treatment completely by impairing the activation of CD8^+^ T cells. Notably, knockout of another IPMG, *CLEC4D*, a C-type lectin receptor that expressed on myeloid cells, also reduced the effect of anti-PD-1 treatment potentially through maintaining the immunosuppressive effects of myeloid cells. Our results suggest that priming TIME via activating the IPMGs may increase the response rate and the effect of immune checkpoint blockers.

## Introduction

The immune system protects the host against tumorigenesis by identifying and eliminating cancerous cells. Under the extreme selective pressure from the host immune system, tumor cells undergo rapid evolution and eventually escape immune surveillance through different mechanisms^1–3^. To sensitize the host immune response against tumor cells, several immunotherapies have been developed over the last three decades, including cytokine therapies, vaccines, and cellular therapies such as chimeric antigen receptor (CAR)-modified T-cell therapy and adoptive cell transfer therapy^4^. Recently, immune checkpoint blockade (ICB) therapies, such as CTLA-4 and PD-1, have achieved unprecedented success in treating advanced melanoma, non-small cell lung cancer, and many other cancer types^5,6^. However, effective responses were only observed in a small subset of patients owing to a high rate of resistance to checkpoint inhibitors among tumors^7^. The disparity in clinical outcomes highlights the phenotypical and functional heterogeneity among different tumors^8^ and among their immune microenvironments^9^. Therefore, to improve the efficacy of immunotherapies, it is imperative to understand the underlying mechanisms of immunotherapy resistance and to develop more reliable prognostic strategies.

Several features are employed by models predicting patient responses to immunotherapy. For example, tumor mutational load and neoantigen load in tumor cells correlate with prognostic outcome for ICB^10,11^. In addition, many signaling pathways are identified as predictive biomarkers for tumor sensitivity to ICB, e.g., chronic type I and type II interferon (IFN) signaling^12^, phosphatase and tensin homolog (PTEN)-related oncogenic pathways^13^, and oxidative stress-related metabolic processes^14^. Features of the tumor immune microenvironment (TIME), such as the interaction of nature killer–dendritic cell (DC) axis^15^, the enrichment of CD8^+^ T cells, the presence of the galectin-9^+^ DC/DC-like macrophages, and a high M1/M2 macrophage ratio^16^ were also reported to be associated with the response to immunotherapy. Recently, a computational method showed that the expression signatures of T-cell dysfunction and exclusion predicted ICB response with high accuracy^17^.

However, previous efforts to identify effective prognostic biomarkers for immunotherapies usually focus on only one type of immunotherapy or limited to one type of cancer and suffer from low statistical power, owing to the limited number of patients involved in each of the studies. Here we sought to increase the statistical power of detecting genes related to patient survival by combining information from different studies involving various immunotherapies. Despite targeting distinct immune modulators, the common goal of the different immunotherapies is to eliminate tumor cells indirectly by promoting immune responses. Recently, single-cell RNA sequencing (RNA-seq) analysis showed that tumor cells had a high degree of inter-tumor heterogeneity; however, tumor-infiltrating immune cells were clustered by cell types independent of samples and showed highly homogeneous across cancer patients^18,19^. Given the invariant nature of immune cells among cancer patients and the success of immunotherapies across cancer types with distinct tissue origins^5,6^, it is possible that the outcomes of treatments depend largely on the sensitivity of the TIME to the therapeutic stimulus and not the cancer type. Under the assumption that immune responses are independent of the tissue of origin of the tumor, we can pool patients receiving immunotherapies from various sources to increase our chance of discovering predictive pan-cancer biomarkers.

**Fig. 1:**
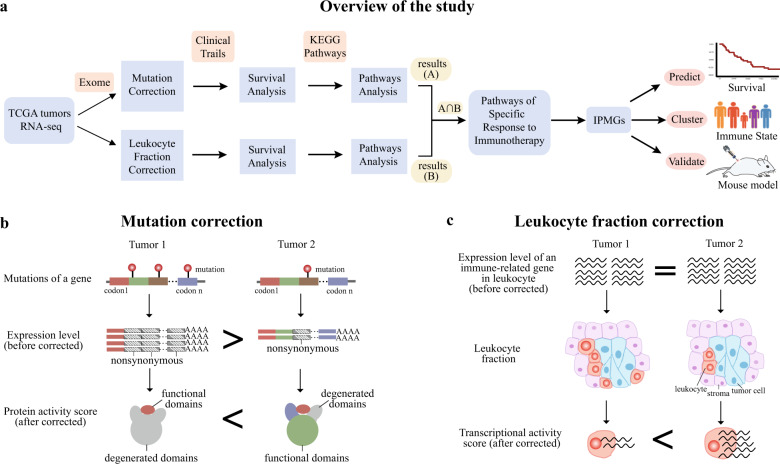
Mutation and leukocyte fraction correction. **a** The schematic workflow of our study. **b** Diagram of “mutation correction” that accounts for the functional reduction of protein-coding genes caused by non-synonymous mutations. The gene in the example is more active in tumor 2 than in tumor 1, despite being more highly expressed in tumor 1 due to multiple non-synonymous mutations impairing the function of the protein in tumor 1. **c** Diagram of “leukocyte fraction correction” that removes the confounding effect of immune cell proportions on the measuring activity of immune-related genes with RNA-seq. Although the same number of total transcripts of a gene are detected in the two tumors, the gene is more actively transcribed on average in tumor 2 than in tumor 1 due to a smaller number of tumor-infiltrating immune cells in tumor 2.

Here we aimed to identify a panel of genes to predict patient survival after immunotherapy by integrating multi-dimensional data of 33 cancer types characterized by The Cancer Genome Atlas (TCGA) (Fig. 1a). To represent the functionality and activity of the genes more accurately, we developed two strategies to adjust the expression table composed of 11,069 pre-treatment tumor biopsy samples. One strategy is defined as “mutation correction,” which measures the functional activity of a gene involved in cancer progression by incorporating gene mutation information and gene expression at mRNA level (Fig. 1b). The other strategy is defined as “leukocyte fraction correction,” which uses the fraction of immune cells to scale gene expression levels for a more precise evaluation of the activity of immune-related genes (Fig. 1c). We then applied survival analysis and pathway analysis to these two adjusted expression tables separately, to find key pathways associated with the survival of patients receiving immunotherapies. Utilizing immunotherapy prognostic marker genes (IPMGs) in the identified pathways, we can predict the outcome of patients treated with distinct immunotherapies in both the TCGA cohort and two independent datasets with high accuracy, highlighting that the state of TIME is associated with its sensitivity to various therapies. Finally, we validated that two of the IPMGs, an essential gene for T-cell receptor signaling (*MALT1*) and a myeloid cell surface receptor (*CLEC4D*), are required in the response to immunotherapy using mouse models.

## Results

### Pathways associated with the clinical outcome of immunotherapy

The definition of therapeutic responses to treatment differs significantly among cancer types and various treatments. To combine information from all 2836 patients whose clinical data and RNA-seq were available in the TCGA cohort treated with different therapies, we used patient survival as an unbiased measure of the effectiveness of the treatments. Less than 2% of the patients included in the TCGA cohort received immunotherapies such as cytokines, cancer vaccines, anti-CTLA-4 therapy, or other monoclonal antibodies. To overcome the limited statistical power in identifying genes correlated with patient survival after immunotherapy, we combined patient data across cancer types and various immunotherapies under the assumption that the immune system’s response to cancer immunotherapy is largely determined by the sensitivity of TIME. We included 99 patients receiving immunotherapies across 12 cancer types, considering the availability of transcriptomic data and clinical information necessary to perform the downstream analysis (Supplementary Data 1).

**Fig. 2:**
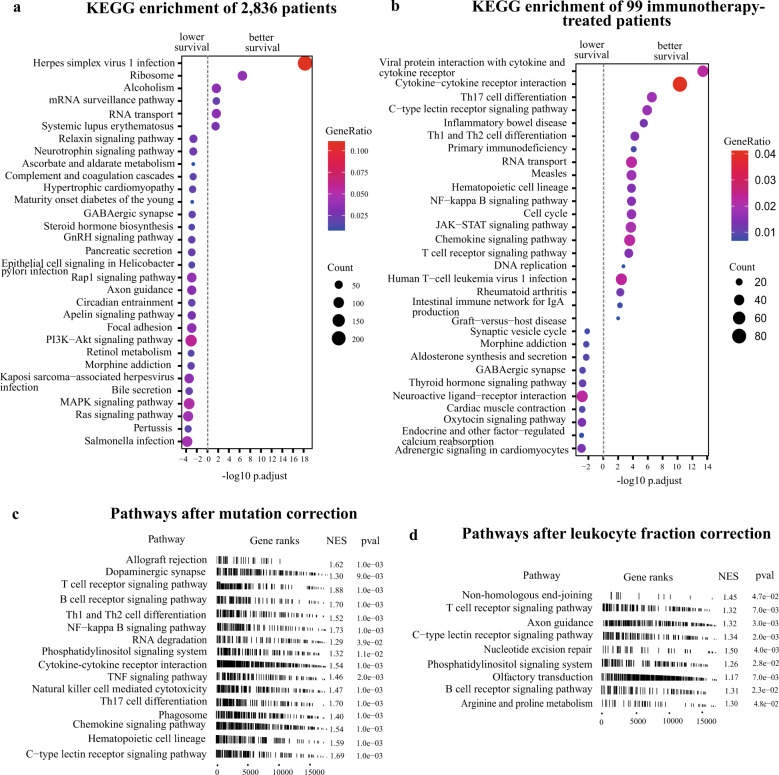
Pathways of response to immunotherapy. **a** KEGG enrichment of genes of which higher expression were associated with lower (left) or better (right) survival of all 2836 patients for which clinical information was available based on expression profile without any correction (log-rank test, FDR adjusted *p*-value < 0.05). **b** KEGG enrichment of genes of which higher expression were associated with lower (left) or better (right) survival of 99 patients receiving immunotherapy based on expression profile without any correction (log-rank test, FDR adjusted *p*-value < 0.05). **c**, **d** Pathways associated with immunotherapy-specific responses based on “mutation correction” profile (**c**) and “leukocyte fraction correction” profile (**d**). Only pathways with a statistically significant importance score after feature selection are shown here.

For every gene in the human genome, we first evaluated the association between its expression in the tumors and the survival of all the 2836 patients regardless of the treatment strategies. Kyoto Encyclopedia of Genes and Genomes (KEGG) enrichment analysis showed that genes of which higher expression was significantly associated with lower survival were enriched in a variety of oncogenic signaling pathways, such as phosphatidylinositol-3-kinase (PI3K)-Akt, mitogen-activated protein kinase, Ras signaling, and focal adhesion (Fig. 2a). The expression of oncogenic signaling genes is indicative of tumor characteristics such as cancer type, progression, stage, or metastasis; therefore, it is usually associated with patient survival regardless of the treatment strategies. Few pathways were enriched for genes of which higher expression was associated with improved survival of all patients (Fig. 2a). When applied the same analysis on the 99 patients receiving immunotherapy, we found that genes of which higher expression was significantly associated with improved survival were mainly enriched in immune-related pathways (Fig. 2b), suggesting that the survival of these patients depends not only on the level of tumor progression but also on the sensitivity of TIME to immunotherapy. Immune-related pathways have a considerable impact on the survival of patients receiving immunotherapy.

One caveat of using patient survival as an indicator for the efficacy of immunotherapy is that it is confounded by tumor characteristics that are generally correlated with patient survival regardless of the treatment strategy. Immunotherapy-specific pathways are more appropriate predictors for immunotherapy efficacy and thus more desirable in the clinical setting. To determine whether a pathway can serve as an immunotherapy-specific predictor, we performed pre-ranked gene set enrichment analysis (GSEA)^20^ on patients receiving immunotherapies controlled against patients treated with non-immunotherapies. Pre-ranked GSEA analysis showed that 55 and 32 pathways were significantly enriched after mutation correction (Fig. 2c) and leukocyte fraction correction (Fig. 2d), respectively, whereas only 22 pathways were enriched without any correction of the gene expression table (Supplementary Data 2). Reassuringly, analysis with the two expression–correction strategies recovered pathways known to be related to immunotherapies, such as T-cell receptor and B-cell receptor pathways, whereas analysis without correction showed no enrichment in the known immune-related pathways, suggesting that both corrections significantly reduced the effect of the confounders in the data as intended.

A majority of the immunotherapy-associated pathways we identified were immune system-related and only a fraction of them were oncogenic signaling pathways (Supplementary Data 2), suggesting that they are indeed immunotherapy-specific, i.e., the activities of the pathways have a stronger impact on immunotherapy beyond their general association with the characteristics of the tumors. Interestingly, we observed enrichment in pathways linked not only to the adaptive immune system but also to the innate immune system, the function of which is not yet the main focus of current immunotherapies.

### The gene panel from the identified pathways can predict patient survival after immunotherapy

**Fig. 3:**
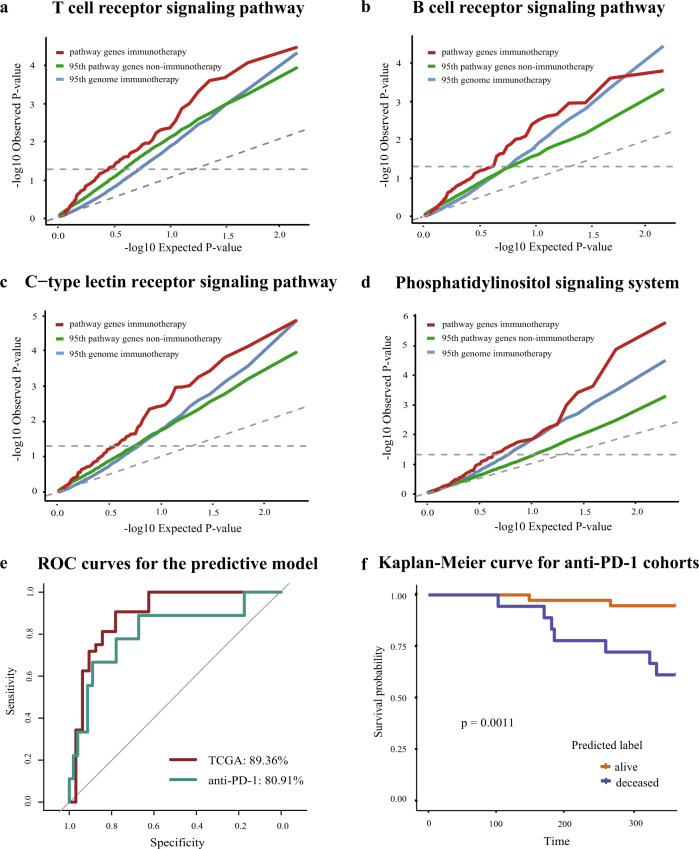
The gene panel from four pathways predicts patient survival following immunotherapy. **a**–**d** Quantile–quantile plots showed that the *p*-value distribution in the survival analysis of pathway genes in patients treated with immunotherapy (red line) was more significant than that of patients treated with other therapies (green line). The 95th percentile of *p*-value from the survival analysis of random genome after immunotherapy was plotted as the background control (blue line). **e** Performance of the logistic regression model built with the 40 IPMGs in different cohorts. **f** Kaplan–Meier plot shows a significant difference in survival (the log-rank test, *p*-value < 0.05) between alive and deceased groups predicted by the elastic net logistic regression model in the anti-PD-1 therapy-treated cohorts.

Biomarkers currently used to screen patients for costly immunotherapies in the clinic are inadequate, leading to a low response rate among patients receiving the treatments. We sought to test whether the expression of immunotherapy-specific genes in pre-treatment tumor biopsies can predict patient survival accurately and whether these genes can serve as potential biomarkers for clinical use. Requirements of a practical patient-screening procedure include low cost and rapid assessment; therefore, we first reduced the number of pathways needing to be tested, to make an accurate prediction. To find the pathways with the greatest bearing on predictive power, we performed feature selection using the random survival forest (RSF)^21^ approach, which gives priority to features in the survival analysis. After RSF ranking, we chose 16 out of 55 pathways enriched after mutation correction and 9 out of 32 pathways enriched after leukocyte fraction correction based on feature importance (RSF variable importance, *p*-value < 0.05) (Supplementary Data 3). Four pathways consistently shown to be enriched after both corrections were the T-cell receptor signaling pathway, the B-cell receptor signaling pathway, the C-type lectin receptor (CLR) signaling pathway, and the phosphatidylinositol signaling system. We observed the same trend of enrichment in the quantile–quantile plot (QQ plot) (Fig. 3a–d and Supplementary Fig. 1a–d), which compares the association between gene expression and patient survival after immunotherapy vs. non-immunotherapy.

The T-cell and B-cell receptor signaling pathways are well-known to play a crucial role in tumor immunity^22,23^ and their regulation mechanisms in the tumor microenvironment have been intensively investigated over the years^24,25^. However, the functions of the CLR signaling pathway and the phosphatidylinositol signaling system in tumor immunity are not well understood. The CLR signaling pathway is mainly involved in complement activation, phagocytosis, and innate immunity^26^. By specifically recognizing glycans, CLRs may participate in the direct interaction between tumor cells and immune cells, and facilitate tumor rejection^27^. The phosphatidylinositol signaling system is an intricate network of kinases and phospholipid messengers that tightly controls many cellular processes such as cell signaling and metabolic regulation. The enzymes PI3K and PTEN, which regulate phosphatidylinositol-3,4,5-trisphosphate play important roles in cancer development^28^.

Among genes in the four pathways, we identified 64 candidate genes that were significantly related to patient survival (Supplementary Data 4). We next examined whether expression levels of the 64 IPMGs could predict the survival of patients after immunotherapy. We used TCGA data as the discovery set to build an elastic net logistic regression model and then tested the accuracy of the model on the data of two independent cohorts of patients who had received anti-PD-1 therapy^7,29^, to evaluate the generalizability of the model. Cross-validation in the TCGA cohort showed that the model taking the expression of the top 40 IPMGs as input achieved the highest 88.91% accuracy and an area under the receiver operating characteristic curve (AUC) of 89.36% in the TCGA test data (sensitivity = 85.94%, specificity = 91.88%) (Supplementary Fig. 1e). Surprisingly, even though no patient was treated with anti-PD-1 in the TCGA discovery cohort, the model achieved a mean accuracy of 70.54% (sensitivity = 85.52%, specificity = 55.56%) and an AUC of 80.91% (Fig. 3e) in the two independent anti-PD-1 cohorts. Consistently, patients classified to alive and deceased groups by the model displayed a significant difference in survival (Fig. 3f). Furthermore, The IPMGs also showed comparable results as other published biomarkers using the biomarker evaluation module tool^30^ (Supplementary Fig. 2a–c). These results suggest that the expression of the 40 IPMGs can serve as a set of pan-cancer prognostic biomarkers for immunotherapies.

### The expression of IPMGs can reflect the sensitivity of TIME to immunotherapies

**Fig. 4:**
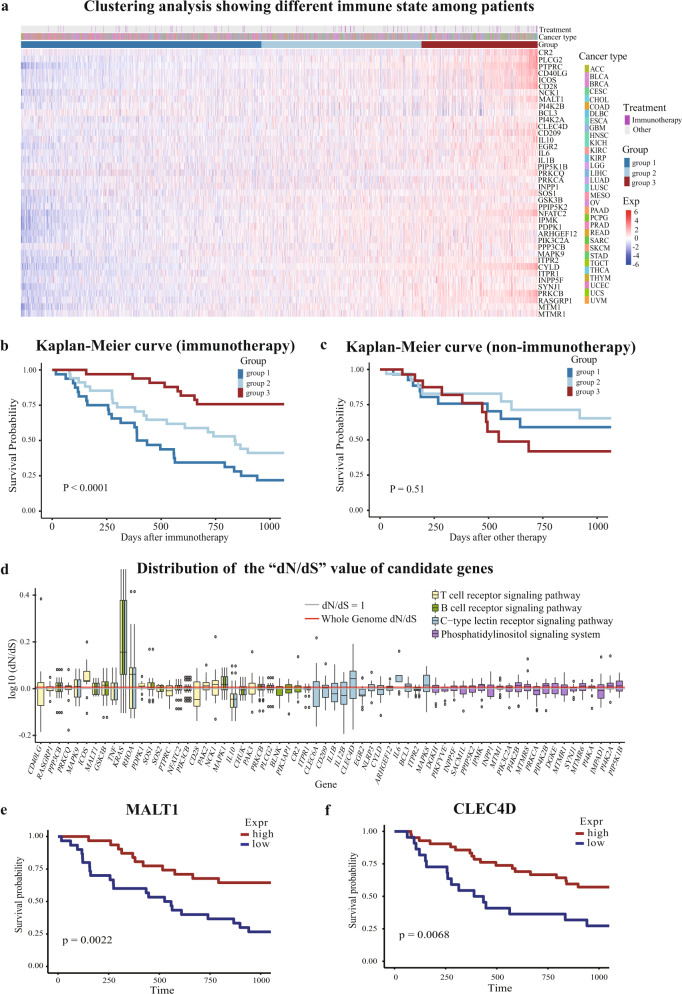
IPMGs define the state of TIME across cancer types. **a** Clustering of patients based on the ssGSEA score of the top 40 IPMGs. A total of 2836 patients across 32 cancer types were clustered into three groups. **b** The survival of patients receiving immunotherapy differed significantly in the three groups. **c** No significant survival difference was observed among patient groups receiving non-immunotherapy. **d** The “*dN*/*dS*” value of IPMGs in four pathways. Each boxplot represents the distribution of “*dN*/*dS*” values across all cancer types for a gene. The *Y* axis was log transformed. Most of the IPMGs are not positively selected in cancer. **e** The expression level of *MALT1* was positively associated with the improved survival of immunotherapy-treated patients based on mutation-corrected expression (the result of fraction-corrected expression was showed in Supplementary Fig. 5d). **f** The expression level of *CLEC4D* was positively associated with the improved survival of immunotherapy-treated patients based on mutation-corrected expression (the result of fraction-corrected expression was shown in Supplementary Fig. 5e).

To assess whether the gene panel comprising the 40 IPMGs reflects the state of TIME rather than the characteristics of specific tumors, we performed a clustering analysis on 2836 patients that have clinical information using the single-sample GSEA (ssGSEA) score^31^. A total of 2836 patients across 32 cancer types were clustered into 3 groups comprising mixed cancer types (Fig. 4a). Interestingly, among the three groups, patients receiving immunotherapy have distinct survival rates (Fig. 4b), whereas patients receiving other treatments do not (Fig. 4c). Differential expression analysis and GSEA revealed that many immune-related pathways, such as antigen processing and presentation, and B-cell and T-cell receptor signaling pathways are activated in the group of patients with prolonged survival after immunotherapy (Supplementary Data 5). These results suggest that these 40 IPMGs can classify cancer patients into groups with distinct TIME (Fig. 4a–c and Supplementary Figs. 3a–f and 4a–f), which may influence the outcome of immunotherapy, but not other treatments.

Unlike the signature genes of tumor progression, in which genetic alterations are usually positively selected during tumor evolution, immune-related genes expressed in immune cells are unlikely to accumulate somatic mutations due to the lack of natural selection on the genetics level of immune cell during tumorigenesis. Indeed, the selective pressure on mutations in the vast majority of the IPMGs during tumor development, measured by the ratio of substitution rate at non-synonymous site and synonymous site (*dN*/*dS*), is similar to the average selective pressure on all genes in the human genome (Fig. 4d). One interesting exception is the well-known oncogene, *KRAS*, which promotes the continuous proliferation of tumor cells after acquiring a gain-of-function mutation. *KRAS* is also found to be involved in tumor immunogenicity^32^, suggesting that our method can detect genes that have confounding effects on patient survival, i.e., genes that are both oncogenic and are related to tumor immune responses. Further dissection of confounded functions of such genes may require the use of single-cell technology to accurately measure the expression of these genes in different types of cells residing in the tumor microenvironment.

Single-nucleotide polymorphism (SNP) sites were found to be associated with the activity of the immune system in previous studies. An SNP can affect the activation and development of CD4^+^ T cell^33^ and an analysis via genome-wide association studies (GWASs) observed six SNPs in the human leukocyte antigen genes linked with vaccine-specific antibody responses^34^. Indeed, our analysis also showed that seven SNP sites in gene-body and up-/downstream 100 kb regions of four IPMGs (*PPP3CB*, *ITPR1*, *PI4K2B*, and *MTMR1*) were significantly linked to the survival of immunotherapy-treated patients (Supplementary Fig. 5a and Supplementary Data 6). These results suggest the genetic diversity of the IPMGs in the population can partly explain the variation in the sensitivity of TIME to immunotherapies.

### Knockout of *MALT1* or *CLEC4D* eliminated the antitumor effect of anti-PD-1 treatment in mouse models

The aforementioned computational analysis shows that the IPMGs we identified can be used as biomarkers for predicting the patient response to immunotherapy. To test whether these results reflect a correlation or causation between the expression of the IPMGs and patient survival (Fig. 4e, f), we used experimental models to further explore the potential roles of the IPMGs in promoting immune clearance of tumor cells during immunotherapy treatment. Specifically, we focused on two IPMGs as follows: (a) *MALT1* represents well-established genes that are critical to receptor signaling in the adaptive immune system and (b) *CLEC4D* represents less understood genes that are mainly expressed on myeloid cells of the innate immune system.

**Fig. 5:**
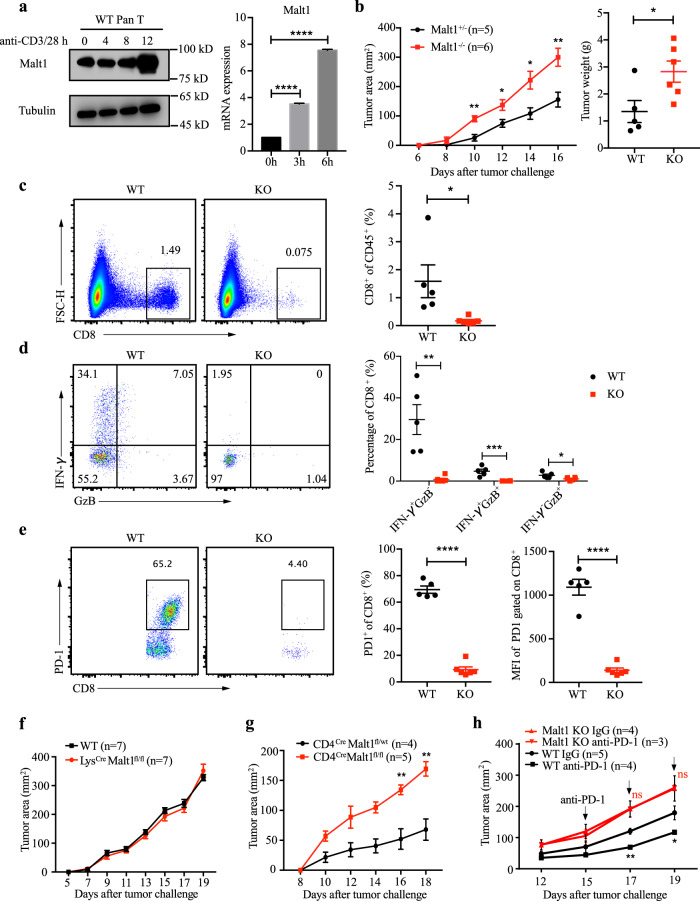
*MALT1* plays a profound role in the immune rejection of tumor cells and the response to the anti-PD-1 therapy. **a** WT Pan T cells were stimulated by plate-bound anti-CD3/28 (5 μg/ml) for the indicated time points. The *Malt1* protein level (left) and mRNA level (right) was detected by WB or qPCR, respectively. **b** Tumor cells were inoculated subcutaneously in *Malt1*^*+/*−^ and *Malt1*^*−/−*^ mice. Tumor growth was measured using calipers at indicated time points and tumor weights were calculated at the end stage. **c** The percentage of CD8^+^ T cells in the tumor-infiltrated lymphocytes (TILs) gated on CD45.2^+^. **d** The percentage of IFN-γ- and Granzyme B-producing CD8^+^ T cells gated on CD8^+^ cells. **e** The FACS plot of PD-1-expressing CD8^+^ T cells gated on CD8^+^ cells, and statistical results of PD-1^+^CD8^+^ percentage and mean fluorescence intensity (MFI) of PD-1 expression on CD8^+^ T cells. **f** Tumor growth in LysCre/*Malt1*^*fl/fl*^ and control mice. **g** Tumor growth in CD4Cre/*Malt1*^*fl/fl*^ and control mice. **h** Tumor growth in mice treated with anti-PD-1 antibody or IgG control. Statistical analysis was performed by unpaired Student *t*-test and the error bars represent ± SEM. *p < 0.05, **p < 0.01, ***p < 0.001, ****p < 0.0001. These results are from one representative experiment of three independent biological replicates.

*MALT1*, also known as paracaspase, can form a complex with CARMA1-BCL10 to mediate T Cell Receptor(TCR)-induced nuclear factor-κB (NF-κB) activation. Upon TCR engagement, its protease activity can also be activated to cleave negative regulators, such as A20 and CYLD, to amplify the NF-κB signaling^35^. Here we sought to explore the role of *MALT1* in the antitumor immune response, which has not yet been elucidated. First, we found *Malt1* was induced upon TCR stimulation in T cells (Fig. 5a), implying that the *Malt1* expression level is indicative of the status of T-cell activation. Moreover, the in vivo tumor model showed that tumor development was promoted in mice deficient in *MALT1* (*Malt1*^*−/−*^)^36^ with increased tumor weight at the end stage compared with wild-type (WT) mice (Fig. 5b). Analysis of tumor-infiltrating lymphocytes showed decreased CD8^+^ T-cell infiltration and IFN-γ or Granzyme B production in *Malt1*^*−/−*^ mice (Fig. 5c, d). PD-1, which could be induced upon T-cell activation, nearly disappeared in *Malt1*-deficient CD8^+^ T cells (Fig. 5e), suggesting defective activation of T cells in mice lacking *Malt1*.

To further determine the cell type(s) in which *Malt1* functions during the antitumor immune response, we crossed *Malt1*^*fl/fl*^ mice^37^ with Lys-Cre or CD4-Cre mice, to specifically delete *Malt1* in macrophages or T cells, respectively. We found that tumor development was enhanced in mice with a specific deletion of *Malt1* in T cells, but not in macrophages (Fig. 5f, g), similar to the phenotype observed in *Malt1* germline knockout (KO) mice, implying that *Malt1* in T cells (Supplementary Fig. 5b) is critical in antitumor immune response. As the survival analysis showed that *MALT1* expression level was positively associated with the improved prognosis of patients after immunotherapy (Fig. 4e), we treated tumor-bearing WT or *Malt1*^*−/−*^ mice with anti-PD-1 to compare with IgG control. We found *Malt1*^*−/−*^ mice failed to respond to PD-1 blockade, whereas the therapy can decrease tumor growth in WT mice (Fig. 5h). This finding was consistent with the observation that *Malt1*-deficient CD8^+^ T cells showed low expression of PD-1 in the tumor microenvironment. Overall, *MALT1* is required for the activation and cytotoxic function of T cells, and the effect of anti-PD-1 therapy is completely abolished in the absence of *MALT1*.

**Fig. 6:**
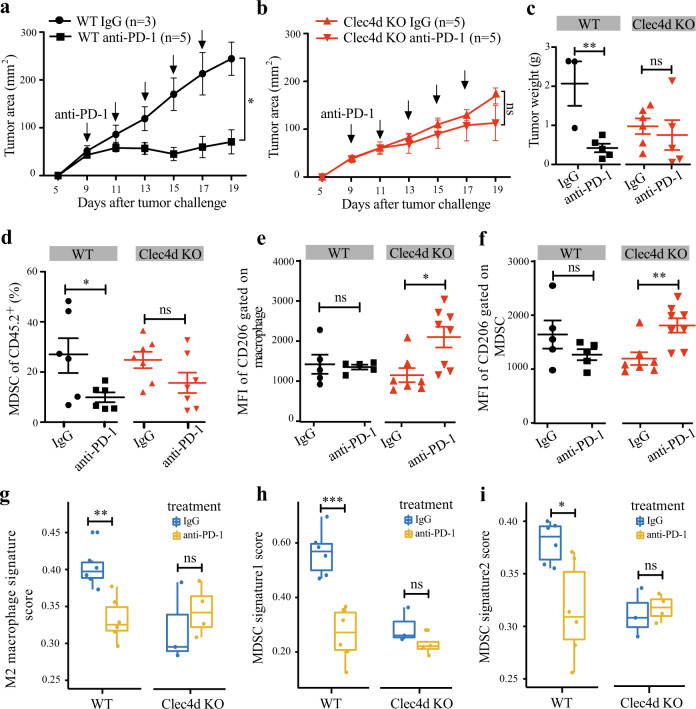
*CLEC4D* affects tumor response to the anti-PD-1 therapy. **a**–**c** WT and *Clec4d*^*−/−*^ mice (**a**, **b**) were implanted subcutaneously with tumor cells. Tumor growth in mice treated with anti-PD-1 or IgG control was measured at indicated time points. Tumor volumes for the last time point were also shown (**c**). **d** The percentage of MDSC in the TIL gated on CD45.2^+^. **e**, **f** The MFI of CD206 on macrophages (**e**) and MDSCs (**f**) in relative subpopulations. **g** The mean expression of signature genes for M2 macrophages from a previous study^45^ in indicated groups. **h**, **i** The mean expression of MDSCs signature genes from previous studies (**h**) and CellMarker database (**i**) in indicated groups. Statistical analysis was performed by unpaired Student *t*-test and the error bars represent ±SEM. *p < 0.05, **p < 0.01, ***p < 0.001. Data from one representative experiment of three independent experiments is shown.

Next, we chose to explore the function of *CLEC4D* (also called Dectin-3, CLECSF8, or MCL), a CLR that is well-known to mediate anti-fungal innate immune responses^26^, which showed the same positive effect on the immunotherapy outcome as *MALT1* (Fig. 4f). Consistent with our computational analysis, *Clec4d*^*−/−*^ mice^38^ receiving anti-PD-1 treatment showed no significant reduction in tumor burden compared with *Clec4d*^*−/−*^ mice receiving IgG control, whereas the treatment can inhibit tumor development in groups of WT mice (Fig. 6a–c). To further investigate the mechanism underlying the lack of response to anti-PD-1 therapy in *Clec4d*^*−/−*^ mice, we analyzed the characteristics of the tumor-infiltrating myeloid cells for the reason that *CLEC4D* is mainly expressed on myeloid cells (Supplementary Fig. 5c). In responsive WT mice, we observed that the percentage of myeloid-derived suppressor cells (MDSCs) was markedly reduced (Fig. 6d). The MFIs of CD206^+^ (the marker for pro-tumoral M2 macrophages) expressed on both macrophages and MDSCs were also trending down after anti-PD-1 treatment (Fig. 6e, f), consistent with the view that anti-PD-1 therapy can relieve partial immunosuppression mediated by pro-tumoral M2 macrophages and MDSCs^39,40^. In contrast, the percentage of MDSCs is not significantly reduced in the *Clec4d*^*−/−*^ mice after anti-PD-1 treatment and the MFIs of CD206^+^ expressed on both macrophages and MDSCs were increased (Fig. 6d–f). These results suggest that KO of *Clec4d* limits the efficacy of anti-PD-1 treatment via maintaining the myeloid-mediated immunosuppressive effect.

To further confirm the myeloid-mediated immunosuppression in TIME, we performed RNA-seq experiments on mixed macrophages and MDSCs sorted from tumor tissues in WT and *Clec4d*^*−/−*^ mice with/without anti-PD-1 treatment. Our analysis showed that signature of M2 macrophages was indeed significantly downregulated in WT mice upon anti-PD-1 treatment, but such signature was trending up, although not statistically significant up in *Clec4d*^*−/−*^ mice upon treatment (Fig. 6g–i). Similarly, two separate sets of markers for MDSCs were both significantly downregulated in WT mice upon anti-PD-1 treatment; however, no significant difference is observed in *Clec4d*^−/−^ mice. Altogether, these results suggest an essential role of *CLEC4D* for myeloid-mediated immunosuppression in limiting the efficacy of anti-PD-1 therapy, highlighting the possibility of combinatory strategies by stimulating *CLEC4D* together with anti-PD-1 treatment to improve therapeutic efficiency.

## Discussion

Despite the rapid growth of multi-dimensional omic data derived from tumor samples, the statistical power to identify prognostic markers for cancer immunotherapies is often limited due to the small sample sizes of the individual clinical studies. Under the assumptions that diverse immunotherapies share the common goal of reactivating the host immune system against tumor cells and therefore their effect rely largely on the sensitivity of TIME and not the tumor’s tissue of origin, we integrated multi-dimension data from patients across all cancer types in TCGA, to identify novel biological pathways associated with patient response to immunotherapies. The degree of tumor malignancy and the sensitivity of TIME are key factors impacting the survival outcomes of immunotherapies. However, without controlling the general characteristics of tumors, traditional analysis often detected signatures involved in the transformative, proliferative, and metastatic capabilities of tumors acquired during progression^7^. By selecting pathways that have a stronger correlation with patient survival after immunotherapy than after non-immunotherapeutic treatment, we successfully discovered prognostic genes and pathways that are immune-related and specifically associated with the efficacy of immunotherapy. Using the expression level of IPMGs as features, we clustered patients across multiple cancer types into groups with distinct response rates to immunotherapies and trained a logistic regression model to predict patient survival. Notably, the prediction model achieved high accuracy in patients treated with anti-PD-1 therapy in two independent clinical studies, despite that the discovery data used to train the model consists only patients receiving other types of immunotherapy, suggesting that patients who respond to one type of immunotherapy are likely to benefit from other types of immunotherapies as well. The accurate cross-immunotherapy prediction in pan-cancer analysis supports the assumption that the sensitivity of TIME is likely to be one of the major determinants of the efficacy of a variety of immunotherapies.

Expectedly, the majority of IPMGs positively correlated with response to immunotherapy are pro-inflammatory cytokines (*IL6* and *IL1B*), T-cell co-stimulators (*ICOS*, *CD40LG*, and *CD28*), and positive regulators involved in promoting immune responses against tumors (*NFATC2*, *PRKCQ*, and *NCK1*). It is likely to be that TIME with higher levels of expression of such immune-stimulatory genes is more readily activated after immunotherapy, resulting in a better response. Interestingly, a small number of IPMGs have been reported to suppress immune activation, such as *IL10*^41^ and *CYLD*^42^, indicating that the therapeutic effect of immunotherapies might be achieved by targeting pathways associated with these immune-inhibitors, similar to the effect of the anti-PD-L1 inhibitors to the PD-L1-positive tumors.

Tumor tissue is a mixture of many cell types, including not only tumor cells but also cells residing in the tumor microenvironment, such as various lymphocytes and stromal cells. Here we removed the effect of leukocyte proportion on the expression of immune-related genes and focused on the activity status of the genes. It should be noted that the immune cell composition of the tumor microenvironment can also serve as a prognostic marker for immunotherapies^43,44^. With the rapid development of single-cell RNA-seq technology, dissecting the cell composition of the tumor microenvironment has been made possible^45^. Studying the expression of IPMGs in a variety of functional subpopulations at a single-cell scale would greatly expand our understanding of the mechanisms underlying their function in the immune response to cancerous cells.

The role of adaptive immunity in antitumor response has been substantiated in many studies. Recently, increasing evidence supports that innate immunity also plays a significant role in suppressing growth and progression of malignant tumors. One of the best-studied effectors of innate immunosurveillance is the natural killer (NK) cell. The production of IFN-γ in NK cells induces M1 macrophages, which can manifest the activity of cancer immunoediting in the absence of adaptive immunity^46^. In our study, we found the CLR signaling pathway was linked to response to immunotherapy. Previous research has generally focused on its function in innate recognition of pathogen-associated molecular patterns. One recent study showed that agonists or antagonists of CLRs signaling are potential therapeutic reagents for cancer immunotherapy^27^. Here we validated *CLEC4D*, a member of the CLR superfamily, was critical in mediating the immunosuppression effect of myeloid cells and in tumor resistance to anti-PD-1 therapy. Our results suggest that these IPMGs are not merely biomarkers and reactivating IPMGs in patients with suppressed immune microenvironment may improve the response rate and effect of immune checkpoint blockers.

## Methods

### Molecular and clinical data

We downloaded the gene expression table of the tumor samples from the TCGA cohort generated by the PanCancer Atlas Consortium (https://gdc.cancer.gov/about-data/publications/pancanatlas). A total of 11,069 samples comprising 33 diverse cancer types were included. We replaced the missing values in the expression table with the average expression of the gene in the other samples of the same cancer type. A total of 20,256 genes remained after we excluded genes with expression value <1 in all samples. Finally, the expression table was log_2_ transformed, followed by quantile normalization.

We downloaded mutation annotation files (MAFs) for TCGA patients processed using the VarScan2 pipeline. The MAFs contain detailed information about the locations and the variant types of somatic point mutations in 9850 tumor samples across 33 cancer types.

Clinical record data were downloaded through the TCGA portal. Among the 4298 samples with complete clinical information, we identified 218 patients who had been treated with immunotherapies (Supplementary Data 1).

### Mutation correction

To more accurately represent the functionality of genes in each sample, we adjusted the expression table according to “mutation correction” strategy, which considers the functional disruptions in protein-coding genes due to non-synonymous mutations. Cancer cells accumulate thousands of mutations during the process of tumorigenesis. The functions of the genes driving cancer initiation, progression, and immune evasion are frequently disrupted by non-synonymous mutations, which alter the amino acid sequences of the encoded proteins. Therefore, when investigating the function of cancer-related genes on their expression levels, it is important to consider the consequences of non-synonymous mutations. For example, tumors carrying disruptive mutations in the *TP53* gene appear to have elevated mutation rates regardless of how high the expression level of the gene^47^. To characterize the protein function of cancer-related genes more accurately, we developed “mutation correction” strategy to account for the functional changes in protein-coding genes caused by non-synonymous mutations (Fig. 1b). These detailed procedures are illustrated in the following steps.

We downloaded TCGA MAFs processed by VarScan2 for all cancer types. These files contain the mutation information for every mutation site in each sample. We considered the six classes of base substitutions, C > A, C > G, C > T, T > A, T > C, T > G, and the immediate 5′- and 3′-bases to each mutated base^48^. Considering the strand symmetry, each mutation site can be assigned to 1 of 192 substitution categories. For each cancer type, we calculated the frequency of each category based on the record of MAFs. We divided the number of observed mutations of each substitution category in a specific cancer type by the total number of mutations in all 192 categories, resulting in the background frequency of each substitution category in a specific cancer type.

For each codon of a gene, there are nine possible substitution types when only considering single-nucleotide mutations and each mutation can be identified as non-synonymous or synonymous mutation according to whether this mutation changed the amino acid. Here we denote the *p*_*α*_*, α* ∈ [1, 2*,…*, 9] as the non-synonymous mutation category frequency, *p’*_*α*_*, α* ∈ [1, 2*,…*, 9] as the synonymous mutation category frequency. Then, we summed all the non-synonymous conditions (*n*_*i*_) and synonymous conditions (*s*_*i*_) for the codon *i* of this gene (total *k* codons in the gene). Finally, we summed up all the codons of the gene *j* and calculated the non-synonymous mutation background (*N*_*j*_) and synonymous mutation background (*S*_*j*_).

1 $$n_i = \mathop {\sum }\limits_\alpha p_\alpha \left( {\alpha \in \left[ {1,2, \ldots ,9} \right]} \right)$$

2 $$s_i = \mathop {\sum }\limits_\alpha p_\alpha ^\prime \left( {\alpha \in \left[ {1,2, \ldots ,9} \right]} \right)$$

3 $$N_j = \mathop {\sum }\limits_i n_i\left( {i \in \left[ {1,2, \ldots ,k} \right]} \right)$$

4 $$S_j = \mathop {\sum }\limits_i s_i\left( {i \in \left[ {1,2, \ldots ,k} \right]} \right)$$

For each cancer type, we calculated the non-synonymous mutation number *C*_*j*_ and the synonymous mutation number *C’*_*j*_, which belong to the single-nucleotide variation type for each mutated gene *j*. Then, the *dN*/*dS* metric was calculated as follows:

5 $$\frac{{dN}}{{dS}} = \frac{{\frac{{C_j}}{{N_j}} + 1}}{{\frac{{C_j^\prime }}{{S_j}} + 1}}$$

To identify significantly mutated genes, we used the binomial test in each cancer type individually, to identify genes significantly enriched with non-synonymous mutations compared to the expected background mutation rate. The observed values are the synonymous mutation number and non-synonymous mutation number counted from MAFs, whereas the expected values are the background synonymous mutation frequencies and non-synonymous mutation frequencies from step 2. The final significantly mutated gene list is the union of those genes whose *dN*/*dS* values are >1 and the binomial test has a significant *p*-value (*p*-value < 0.05) in each cancer type. Conceivably, KEGG analysis showed that these hyper-mutated genes were enriched in pathways related to oncogenic signaling and tumor progression (Supplementary Fig. 6a).

We defined a protein activity score to correct the raw expression profiles, to represent the functional activity of a protein more accurately. Notably, the protein activity score was aimed to correct those significantly mutated genes illustrated in step 2. We calculated a pan-cancer mutation category frequency considering the imbalance in sample sizes for different cancer types. We multiplied the initial frequency for each mutation category in a certain cancer type with the corresponding sample number and then summed them up, which was denoted as *r*_*j,k*_. Then, we denoted *T*_*j,k*_ as the number of non-synonymous mutations observed across all cancer types for the codon *k* of gene *j*. For a significantly mutated gene, we calculated an index *I*_*j,k*_ for each of the mutated codons as follows:

6 $$I_{j,k} = \frac{{r_{j,k}}}{{T_{j,k}}}$$

The index was then scaled to 0 ~ 1 for all mutated codons. Next, the protein activity score for gene *i* of sample *m* was calculated by multiplying the codon indexes as follows:

7 $$S_{j,m} = \mathop {\prod }\limits_k I_{j,k}$$

Structural variations such as frameshifts or splicing-site mutations were considered to have a loss-of-function effect and the score was assigned a value of 0. Genes with no mutation observed were assigned a score of 1. Finally, we multiplied the score with the expression value to get the mutation-corrected expression table.

### Leukocyte fraction correction

To more accurately represent the activity of genes in each sample, we adjusted the expression table according to “leukocyte fraction correction” strategy, which determines the activity of immune-related genes in tumor-infiltrating leukocytes by removing the effect of leukocyte proportion on gene expression. The tumor microenvironment consists of not only tumor tissue but also normal tissue, stromal cells, and infiltrating lymphocytes, the gene activities of which play a key role in the antitumor immune response. Gene expression levels in TCGA cohort were measured by RNA-seq of bulk tumor tissues from patients. To better characterize the activity status of the immune-related genes in the tumor microenvironment, we corrected the expression level of these genes by removing the confounding effect of the infiltrating leukocyte fraction (Fig. 1c), as follows.

The leukocyte fraction was assessed previously by identifying genomic regions with differential DNA methylation between pure leukocyte cells and normal tissue^49^. The proportion of tumor cells in a tumor sample—tumor purity—was inferred by ABSOLUTE^50^, which takes advantage of the frequency of somatic DNA alterations in the whole-genome sequencing data.

We define immune-related genes to be corrected as those with an expression level that shows a positive correlation with leukocyte fraction but negative correlation with tumor proportion across all tumor samples (Pearson’s correlation). We identified 676 immune-related genes with a correlation coefficient cutoff of 0.3. Gene ontology enrichment analysis shows that the 676 genes were significantly overrepresented in immune-related biological processes (Supplementary Fig. 6b).

To account for the heterogeneity of the leukocyte proportion between different tumor samples, we calculated the activity of the immune-related genes by dividing the gene expression value by the leukocyte fraction (Fig. 1c).

### Survival analysis

We define the survival time of a patient as the time interval between the date of receiving the immunotherapy and the date of the final follow-up. Using a larger range of survival time might introduce more confounding factors in our analysis, e.g., cancer-free patients may die because of natural aging given a long period of time. Given that the majority of the patients receiving immunotherapy are at a later stage of cancer development, we chose to use 3 years as the upper limit of the survival analysis to limit the effect of the confounders and to ensure that there is a sufficient number of patients who survive to downstream analysis. After processing the survival time, 99 out of the 218 patients received immunotherapy, for whom both transcriptomic and clinical information were available. These patients are across 12 cancer types and received the following types of immunotherapies: vaccines (BCG, AE-37, E-75, oncophage, and HSPPC-96), IFN-α, proleukin, IFN-γ, CTLA-4 inhibitor, and other monoclonal antibodies, respectively. Furthermore, 2737 patients without immunotherapy treatment were retained in our analysis using the same selection criteria (Supplementary Data 1).

The association between gene expression and the survival of patients was evaluated by the log-rank test. Our analysis involves patients of diverse cancer types. If we simply pool patients together and divide them into high-expression and low-expression groups, genes expressed specifically in a certain cancer type may be significantly correlated with patient survival, simply because the survival of patients with this cancer type on average is higher or lower compared with other cancer types. To reduce the confounding effect of cancer-specific genes and differences in overall survival rates between cancer types, we selected the top (bottom) 30% of samples in each cancer type based on gene expression and then merged the top (bottom) samples from all cancer types as the high-expression (low-expression) group. Then, we compared the survival rates of high-expression and low-expression groups by a one-tailed hypothesis of the log-rank test, which means that we tested whether higher gene expression associated significantly with better (Supplementary Fig. 6c) or lower (Supplementary Fig. 6d) survival of patients.

The above survival analysis was performed to rank genes from the whole genome based on the log-rank test *p*-value for 99 patients receiving immunotherapies. Two ranked gene lists were generated based on the first hypothesis that higher expression can lead to better prognosis and the second hypothesis that lower expression can lead to better prognosis. Next, we performed the same analysis on 2737 patients not receiving immunotherapy as a control.

### Identification of pathways correlating with immunotherapeutic responses

To find pathways that can potentially impact immunotherapy, we removed the pathways related to the general cancer status, e.g., cancer initiation, cancer types, or cancer stages. A total of 291 pathways from the KEGG database remained after the removal of 37 pathways related to “cancer” or “disease”. The ranked genome lists based on the statistical significance (the log-rank test *p*-value) of survival analysis were used as input in pre-ranked GSEA^20^ to identify significant KEGG pathways involved in response to immunotherapy.

To determine whether a pathway has a specific effect on patients’ response to immunotherapies or it may affect patient survival regardless of the treatment, first we ranked all genes based on their significant level in the survival analysis for patients receiving immunotherapies and then calculated the *p*-value of pre-ranked GSEA for a specific pathway. If the *p*-values of survival analysis of genes belonging to the pathway are significantly skewed to small values according to the pre-ranked GSEA analysis, the pathway is considered as a survival-related pathway. Next, we randomly sampled the same number of patients treated with non-immunotherapies and performed pre-ranked GSEA as we did for patients receiving immunotherapies. For each pathway, we performed random sampling 1000 times and generated the *p*-value distribution of 1000 pre-ranked GSEA tests on patients receiving non-immunotherapies. If the *p*-value for patients receiving immunotherapies is more significant than the *p*-values for patients receiving non-immunotherapies, we considered the pathway as having an immunotherapy-specific effect on patient survival. Here we performed the analysis on each survival-related pathway. If a pathway has a general effect on patients receiving non-immunotherapies, it is unlikely to have less specific effect on patients receiving immunotherapy. Thus, we only considered one tail, i.e., a pathway has a specific effect on immunotherapy in addition to its general effect, and set the *p*-value rank among 1000 *p*-values of permutations cutoff at the 95th percentile. We consider the analysis on different pathways independent as each pathway is comparing to a background *p*-value distribution that is specific to the pathway. We note that immunotherapy-specific pathways may also have an effect on survival of patients treated with non-immunotherapies, although the significance is less that on survival of patients receiving immunotherapies. Pre-ranked GSEA was performed using by “fgsea” R package^51^.

### Quantile–quantile plot

We validated the results of pre-ranked GSEA by the “QQ plot,” a method used to determine whether a pathway is more significant in patients receiving immunotherapy than patients not receiving immunotherapy. From the comparison of the GSEA results of identified pathways between patients receiving and not receiving immunotherapy, we could conclude that these pathways are more significantly enriched in the patients receiving immunotherapy. The QQ plot provides a stricter method to visualize this relationship.

First, we performed survival analysis for patients receiving immunotherapy, then we calculated a *p*-value (log-rank test) for each gene in the whole genome as “*P*-value list A”. Second, we performed the same survival analysis for patients not receiving immunotherapy for 1000 times; for each time, we randomly picked the same patient number as patients receiving immunotherapy and then we calculated 1000 *p*-values for each gene in whole the genome, and nominated this *P*-value matrix as “*P*-value matrix B”. In our analysis, we compared the trend of significance among the three groups of *p*-value for each pathway found in our study. When we sought to study a pathway that contained 50 genes, the first group of 50 *p*-values was derived from “*P*-value list A”, which showed 50 *p*-values for these 50 genes; the second group of 50 *p*-values was derived from “*P*-value list A”, which showed the 95th quantile of randomly selected 50 *p*-values from “*P*-value list A” 1000 times; and the third group of *p*-values was derived from “*P*-value matrix B”, which showed the 95th quantile of these 50 genes based on randomly selected patients not receiving immunotherapy for 1000 times. The first group indicated the trend of significance of the genes on specific pathways in patients receiving immunotherapy, the second group indicated the trend of significance of whole-genome level in patients receiving immunotherapy, and the third group indicated the trend of significance of whole-genome level in patients not receiving immunotherapy. We set two criteria to decide whether a pathway is significantly enriched in patients receiving immunotherapy: the survival significance level of genes of patients accepting immunotherapy on this pathway (the first group) should be higher than the whole genome of patients receiving immunotherapy (the second group); the other should be higher than that of patients not accepting immunotherapy (the third group).

### Feature selection to prioritize pathways and IPMGs

Fifty-five (32) pathways specifically correlated with immunotherapeutic responses were identified based on the mutation (leukocyte fraction) correction profile. To prioritize these pathways, we performed the RFS method^21^ using “ranger” R package^52^, a nonparametric and nonlinear approach for the analysis of right-censored survival data that has been used in several risk models and determined to be superior to the traditional Cox proportional model. Each pathway can be scored in each patient receiving immunotherapy by ssGSEA^31^. Then, we used the ssGSEA score as the predictor and the survival time (scaled within 1 year) with the final status of patients as response variables, to rank pathways by the feature importance of the RSF output. After feature selection, significant pathways were shown according to their variable importance *p*-values. Then, we intersected significant pathways obtained from two correction methods to find pathways significantly related to survival and then we identified genes showing a strong connection to patient survival from these pathways as IPMGs. These genes were also prioritized by RSF.

### The predictive model of patient response to immunotherapy

To examine whether the expression level of candidate genes from the immunotherapy-specific pathways can predict the survival of patients after immunotherapy, we performed elastic net logistic regression to predict the survival status (alive or deceased) of patients using “glmnet” packages in R^53^. In addition to the TCGA cohort, we downloaded three additional published cohorts with transcriptome and clinical records of immunotherapy as independent datasets to build prediction model: cohort 1 from Hugo et al.^7^ and cohort 2 from Riaz et al.^29^.

The prediction model (logistic regression) is a binary classifier system to predict the survival status (alive or deceased) of patients at one time point. We found that few patients in other independent cohorts survived over 3 years and most of them were dead within 2 years. Thus, we decided that the survival time in our prediction model is scaled to 1 year, to maintain a balanced classification of the living status. Finally, we counted the number of patients with transcriptome and clinical records within 1-year survival: 127 from TCGA (alive, *n* = 104; deceased, *n* = 23), 24 from cohort 1 (alive, *n* = 16; deceased, *n* = 8), and 32 from cohort 2 (alive, *n* = 31; deceased, *n* = 1). The mRNA expression level is given by fragments per kilobase of transcript per million mapped reads in all samples.

We built different prediction models by selecting the expression of different number IPMGs prioritized by RSF as features: all 64, top 50, top 40, top 30, top 20, and top 10 genes (Supplementary Fig. 1e). For each model, the TCGA data were divided into discovery and validation sets (2/3 and 1/3 of samples). However, there were imbalanced classification problems both in the training and validation set. The Synthetic Minority Oversampling Technique^54^, a very popular method to fight imbalanced classification problems by oversampling new samples of the minority class or undersampling samples of majority class using the nearest neighbors, was used to solve the problem that there were more living patients than deceased patients in the training set by “DMwR” R package. We solved the same problem in the validation set by artificially creating a balanced set. We randomly selected the same number of living patients as deceased patients, then these selected living patients and fixed deceased patients were merged into a validation set. We generated 1000 such validation sets by randomly selecting living patients and got the average performance of 1000 tests as the final performance of a model.

The above cross-validation in the TCGA cohort showed the top 40 IPMGs achieved the best performance. To test the generalizability of their predictive value, we used the TCGA cohort as training data, and cohort 1 and cohort 2 as an independent validation set to build the model. The imbalanced classification problems were solved, as above. The AUC was used to assess the performance of the prediction model.

### Clustering analysis of patients exhibiting different immune state

We used the expression of IPMGs that were used in the predictive model to cluster the 2836 patients: 99 immunotherapy-treated and 2737 non-immunotherapy-treated. We ranked the patients based on ssGSEA scores^31^ calculated based on the expression of the 40 IPMGs. The boundary of ssGSEA scores between groups were set to ensure immunotherapy-treated patients were equally split up into groups. Here, all patients were classified into three groups and each group contained 33 immunotherapy-treated patients (Fig. 4a). We performed the same analysis for two and four groups (Supplementary Figs. 3a–c and 3d–f). Then, we compared the 3-year survival differences of patients receiving immunotherapy among the groups. To test whether the patient groupings are based on patient response to immunotherapy and not merely on the overall survival of the patients, we randomly selected the same number of patients that were treated with other non-immunotherapy in each group to perform survival analysis as a control. We repeated this process 5000 times and compared how many times the survival differences of patients under non-immunotherapy are smaller than those under immunotherapy. We showed the median *p*-value of survival differences of 5000 permutations in the survival plot (Fig. 4c).

To demonstrate the robustness of the clustering result, we used another method, hierarchical clustering, to separate the 2836 patients into two groups (Supplementary Fig. 4a) or four groups (Supplementary Fig. 4d). Reassuringly, in both cases, we observed more significant survival differences among groups in immunotherapy-treated patients (Supplementary Fig. 4b, e) than in non-immunotherapy-treated patients (Supplementary Fig. 4c, f).

A total of 2836 patients across 32 cancer types were clustered into groups with distinct survival rates after immunotherapy in the clustering analysis. The next question we wanted to explore further was what factors contribute to the different response status in different groups. Thus, we performed the one-tailed differential expression analysis (Mann–Whitney *U*-test) for all genes in genome and performed pre-ranked GSEA^51^. The ranked genes, of which high expression associated with prolonged survival, were significantly enriched in many immune-related pathways, whereas those genes of which high expression associated with the worst survival outcomes were enriched in few basic pathways (Supplementary Data 5).

### Genome-wide association studies

To discover SNPs that are associated with response to immunotherapy, we obtained genotype data from TCGA Affymetrix SNP Array 6.0 containing 103 immunotherapy-treated tumor samples. For the top 40 candidate genes, a total of 3956 SNPs was identified in the gene-body and 100 kb surrounding region. After removing SNPs with minor allele frequency <5%, the remaining 3050 SNPs were used for genome-wide association analysis (GWAS) using the R package “rrBLUP”^55^. This analysis examines the relationship between the SNP genotype and the patient phenotype (1-year survival status). To adjust the *p*-value of the analysis, the genotype of each SNP was shuffled 1000 times and the 95th percentile ranking among the shuffled test *P*-value was used as a cutoff. Then, 122 SNPs that significantly correlated with patient survival were identified (*p*-value < 0.05, cutoff < 0.05).

To estimate the association between SNPs and gene expression, we calculated the Pearson’s correlation between the expression of candidate gene and each SNP’s genotype near the gene. In this step, the cutoff of the Pearson’s *p*-value was obtained based on 1000 permutations of the genotype data. Finally, 7 of 122 SNPs were significantly associated with the expression of four candidate genes (Pearson’s correlation coefficient > 0.2; *p* < 0.05; cutoff < 0.05) (Supplementary Data 6).

### RNA-seq analysis

Macrophages and MDSCs were sorted from relative mouse tumor (WT mice received IgG: *n* = 6; WT mice received anti-PD-1: *n* = 6; KO mice received IgG: *n* = 3; KO mice received anti-PD-1: *n* = 4) and then mixed for sequencing. The reads were aligned using HISAT2 (version 2.1.0)^56^ and quantified using htseq-count (version 0.11.2)^57^. Then, transcripts-per-millions (TPMs) were transformed by quantile-normalized in WT or KO group. Expression signature score was defined as the average normalized TPM of the signature genes. MDSCs signature genes were from previous studies (h)^58,59^ and CellMarker database (i)^60^.

### Stimulation of Pan T cells

WT Pan T cells were isolated according to the manufacturer’s protocol for the EasySeP Mouse CD90.2 Pos Slctn Kit II (Stemcell) and the isolated cells were stimulated by plate-coated α-CD3/28 (5 μg/ml) at indicated time points. The cells were collected for either western blotting or quantitative PCR (qPCR) to detect Malt1 protein or mRNA level. Cells were lysed in lysis buffer (150 mM NaCl, 50 mM HEPES pH 7.4, 1 mM EDTA, 1% Nonidet P-40, and protease inhibitors) and total lysates were subjected to SDS-PAGE (sodium dodecyl sulphate-polyacrylamide gel electrophoresis) followed by blotting with indicated antibodies Malt1 (Santa Cruz), Tubulin (Santa Cruz), and secondary antibody (Easybio). Total RNA was extracted by Trizol (Invitrogen) and cDNA was synthesized using the RevertAid First Strand cDNA Synthesis Kit (Thermo Fisher). Quantitative reverse-transcription PCR using 2× SYBR Green PCR Master Mix (Genestar) was performed on the ABI 7500 Real-Time PCR system (Applied Biosystems). Results were obtained using the 2^−△△CT^ method (*Malt1* qPCR primer forward: 5′-CACAGAACTGAGCGACTTCCT-3′; reverse: 5′-CAGCCAACACTGCCTTGGA-3′).

### Tumor model

Cancer cell line E.G7-OVA was kindly provided by Dr. Chen Dong Lab (Tsinghua University, Beijing, China). Aliquots of 2 × 10^5^ E.G7-OVA tumor cells were inoculated subcutaneously into the shaved flank of each mouse. Tumor growth was monitored every other day or 3 days using calipers and tumor sizes were calculated using the following formula: length/2 × width/2 × *π*. For isolation of tumor-infiltrated lymphocytes, tumors were digested with 1 mg/ml Type 2 collagenase (Worthington) in the presence of 10 U/ml DNase I for 1 h at 37 °C prior to centrifuge on a 40% and 70% discontinuous Percoll gradient (GE Healthcare). The isolated cells were incubated with antibodies as follows: Fixable Viability Dye eFluor 506 (cat#65-0866-18), APC-eFluor780 anti-CD45.2 (clone 104, cat#47-0454080), FITC anti-CD45 (clone 30-F11, cat#11-0451-82), APC-eFluor780 α-CD8 (clone 53-6.7, cat#47-0081-82), eFluor 450 anti-CD279 (PD-1) (clone J43, cat#48-9985-82), PE α-Granzyme B (clone NGZB, cat#48-8898-82), and PerCP-Cyanine5.5 anti-CD11b (clone M1/70, Cat#45-0112-82) were purchased from eBioscience. APC α-IFN-γ (clone XMG1.2, cat#505810) and BV421 anti-CD206 (clone C068C2, cat#141717) were purchased from Biolegend. FITC α-Ly6G (clone 1A8, cat#551460) and Alexa Flour700 α-Ly6C (clone AL-21, cat#561237) were purchased from BD Biosciences. The stock solutions of antibodies were diluted at 1 : 400. For cytokine staining, cells were stimulated with 50 ng/ml PMA (Sigma) and 500 ng/ml Ionomycin (Sigma) in the presence of GolgiStop (BD Biosciences) for 5 h at 37 °C and stained for cell surface markers followed by fixation/permeabilization and intracellular cytokine staining (BD Biosciences). Samples were analyzed by LSR Fortessa cytometers (BD Biosciences) and the resulting data were analyzed by FlowJo software. To test the effect of PD-1 blockade, 100 μg anti-PD-1 (J43, Bioxcell, Cat#BE0033) antibody were injected intraperitoneally every other day from Day 15 in *Malt1* KO mice, whereas in *Clec4d* KO mice, anti-PD-1 treatment was started from Day 9.

### Ethics approval

The mouse experiments were conducted following the institutional guidelines and were approved by the Institutional Animal Care and Use Committees at Tsinghua University. The transcriptomic and clinical data of patients in the study were publicly available from the TCGA project. This study did not involve any human participants.

### Reporting summary

Further information on research design is available in the Nature Research Reporting Summary linked to this article.

## Supplementary information

Supplementary file

Supplementary Data 1

Supplementary Data 2

Supplementary Data 3

Supplementary Data 4

Supplementary Data 5

Supplementary Data 6

Reporting Summary

## Data Availability

The data generated and analyzed during this study are described in the following data record: 10.6084/m9.figshare.14034656^61^. The RNA sequencing data are openly available in the Gene Expression Omnibus (GEO) repository via the following accession: https://identifiers.org/geo:GSE158056^62^. The following files are publicly available as part of the figshare data record^61^: the clinical data and genotype data of TCGA tumor samples in the files “Table S4_ImmunotherapyPrognosticMarker Genes_list.xlsx”, “TCGA_clinical_rawdata.rar”, “dNdS_value_AllCancerType.tar.gz”, “TableS1_raw_clincaldata_TCGA.xlsx”, and “immunotherapy-SNP-103.txt”; the tumor growth in WT and KO mice in the files “Clec4d_WT-KO_TumorSize.pzf”, “Malt1_WT-KO-TumorSize.pzfx”, and “MeanFluorescenceIntensity.pzfx’; and the gene expression tables of TCGA tumor samples in the files “EBPlusPlusAdjustPANCAN_IlluminaHiSeq_RNASeqV2-v2.geneExp.tsv.gz”, “TCGA_pancancer.FractionCorrection_logqq.exp.txt.gz”, and “TCGA_pancancer.MutationCorrection_logqq.exp.txt.gz”.

## Code availability

Data analysis pipelines were described in detail in “Methods”. In our analysis, statistical methods and data processing were performed by built-in R functions in R version 3.5.1 (https://www.R-project.org/). All R packages used were described in “Methods”.
