# Supplementary material for: Knockout of immunotherapy prognostic marker genes eliminates the effect of the anti-PD-1 treatment
Source: NPJ Precis Oncol. 2021 May 7;5:37. doi: 10.1038/s41698-021-00175-2 (PMC8105367; doi:10.1038/s41698-021-00175-2)
Supplement: Supplementary file 1 — Supplementary file [file 41698_2021_175_MOESM1_ESM.pdf]

Supplementary Figures

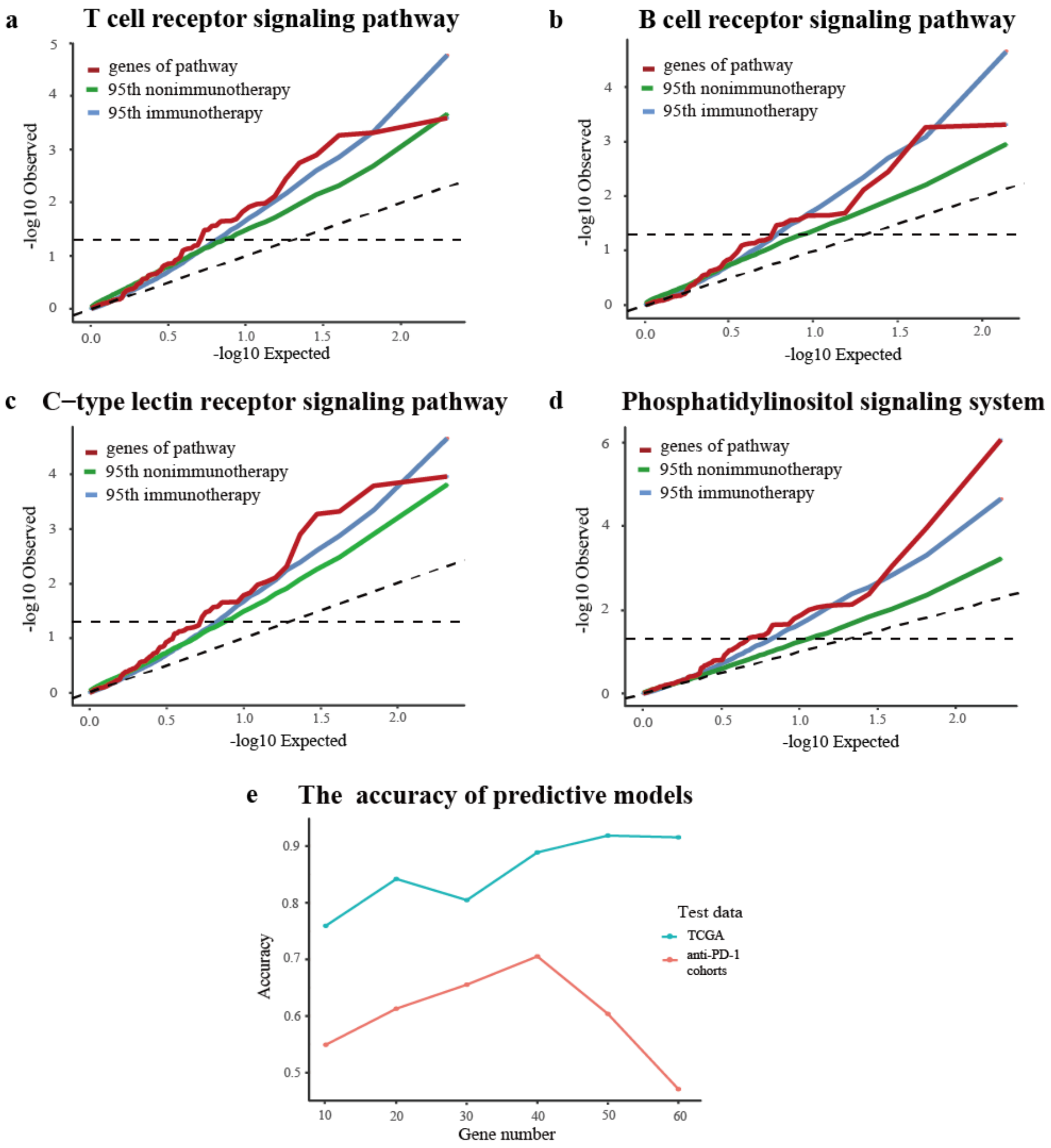

Supplementary Figure 1. Gene panel from four pathways predicts the survival of patients receiving immunotherapy.

a-d Quantile-Quantile plots comparing the distribution of the survival significance of genes from pathway with immunotherapies (red line) and non-immunotherapy (green line) based on leukocyte fraction correction profile. The 95th percentile of survival significance of the random genome after immunotherapy was plotted as a background control (blue line). **e** The IPM gene number effect on the accuracy of logistic regression. Cross-validation in the TCGA cohort via different genes as features by RSF ranking was performed. The model built with the top 40 IPM genes had the best performance both in TCGA cohort and independent anti-PD-1 cohorts.

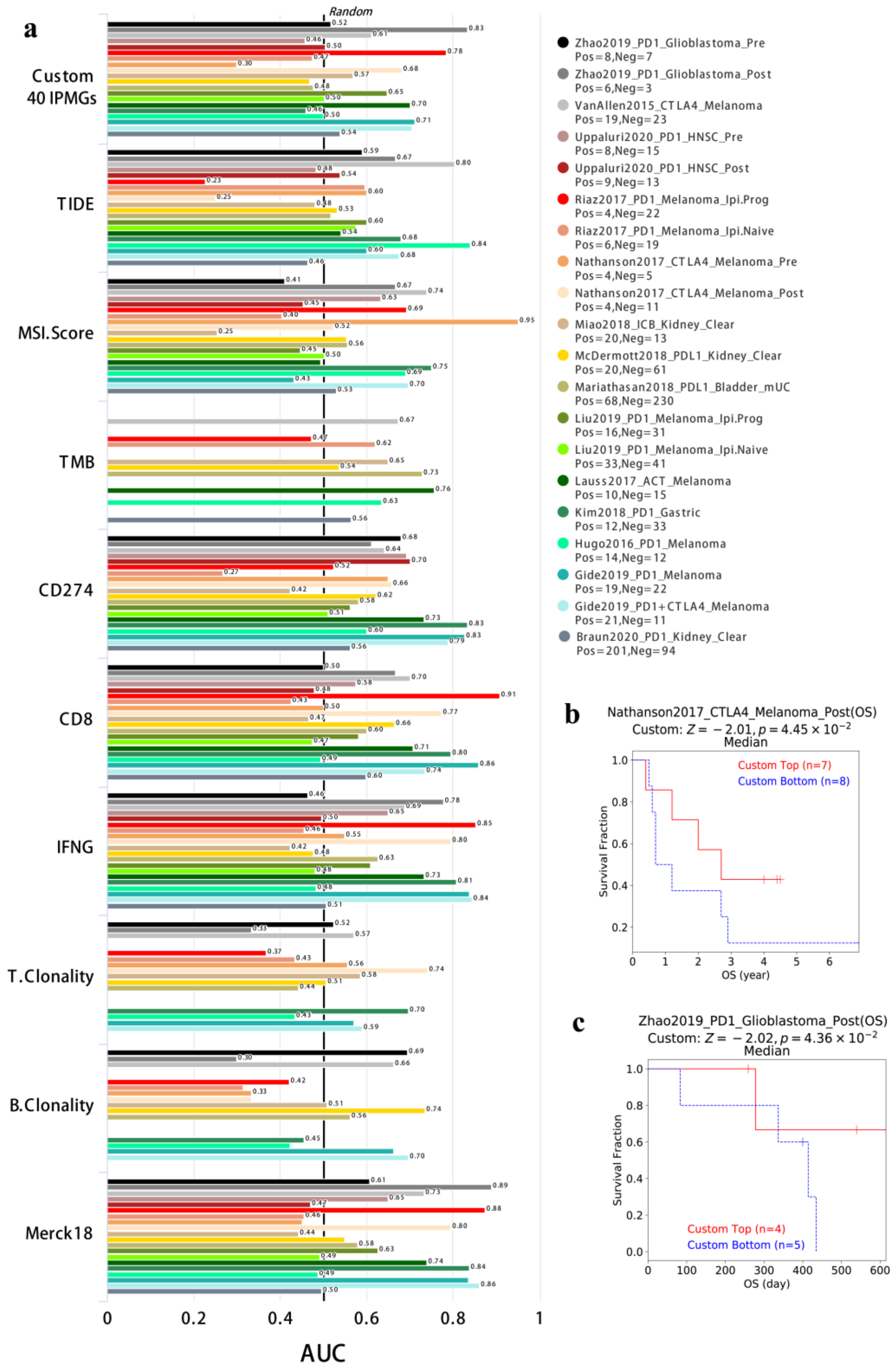

Supplementary Figure 2. Comparison of the predictive power of the IPMGs signature with other published biomarkers.

a Prediction performance of 40 IPMGs on the ICB response status compared with other biomarkers on 20 ICB cohorts (3 sub-cohorts were removed due to less than 30% IPMGs mapped in a total number of 23 sub-cohorts) measured by AUC. **b, c** The association of 40 IPMGs with patients' overall survival through Kaplan-Meier curves. High average expression of the IPMGs showed significant associations with prolonging survival in two sub-cohorts (two-side Cox-PH p-value < 0.05).

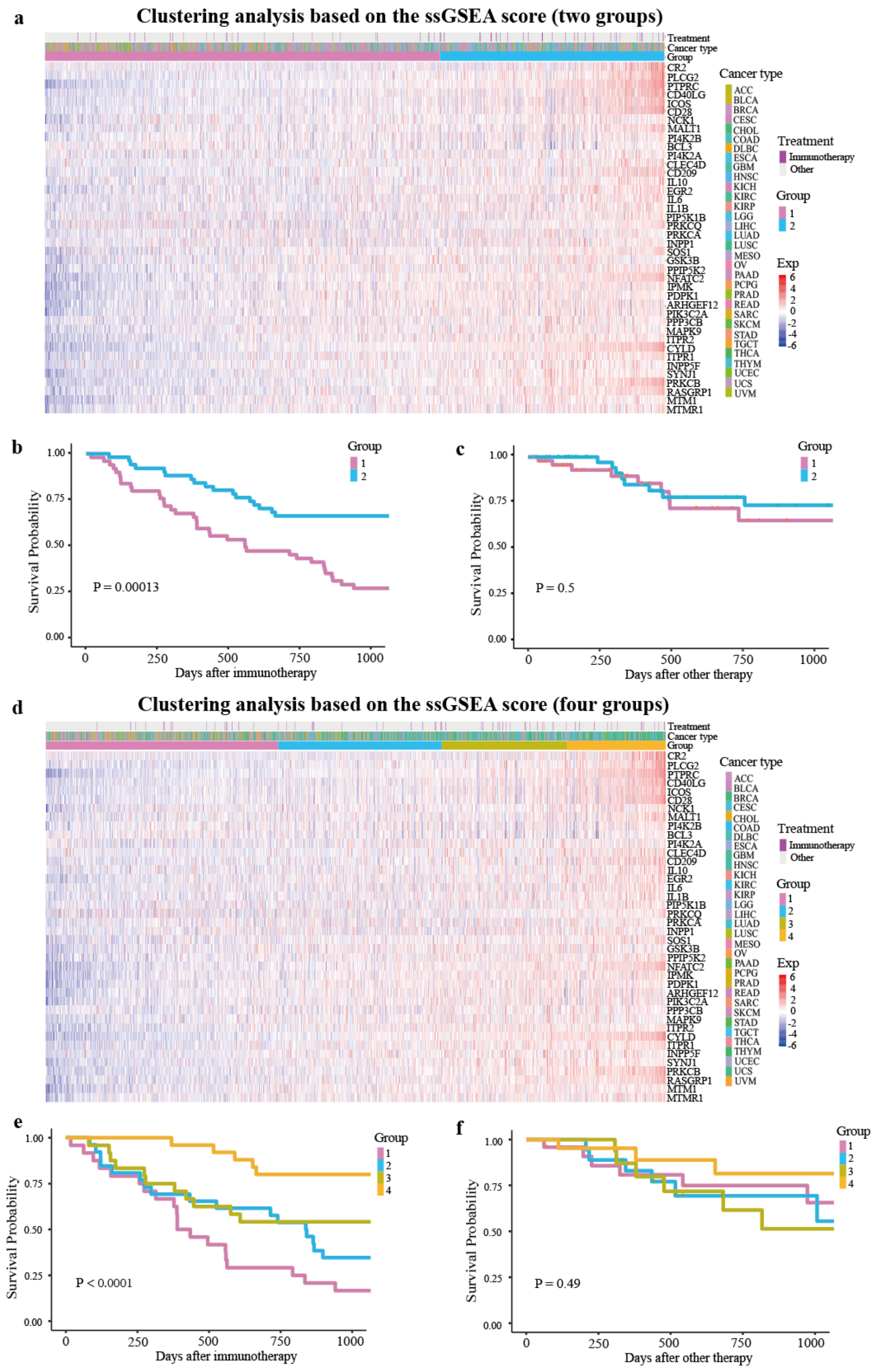

Supplementary Figure 3. Clustering analysis based on the ssGSEA score of the top 40 IPM genes for patients.

a 2,836 patients across 32 cancer types were clustered into two groups. **b** The survival of patients receiving immunotherapy differed significantly in the groups. **c** No significant survival difference was observed among the groups of patients receiving non-immunotherapy. **d** 2,836 patients across 32 cancer types were clustered into four groups. **e** The survival of patients receiving immunotherapy

differed significantly in the groups. **f** No significant survival difference was observed among the groups of patients receiving non-immunotherapy.

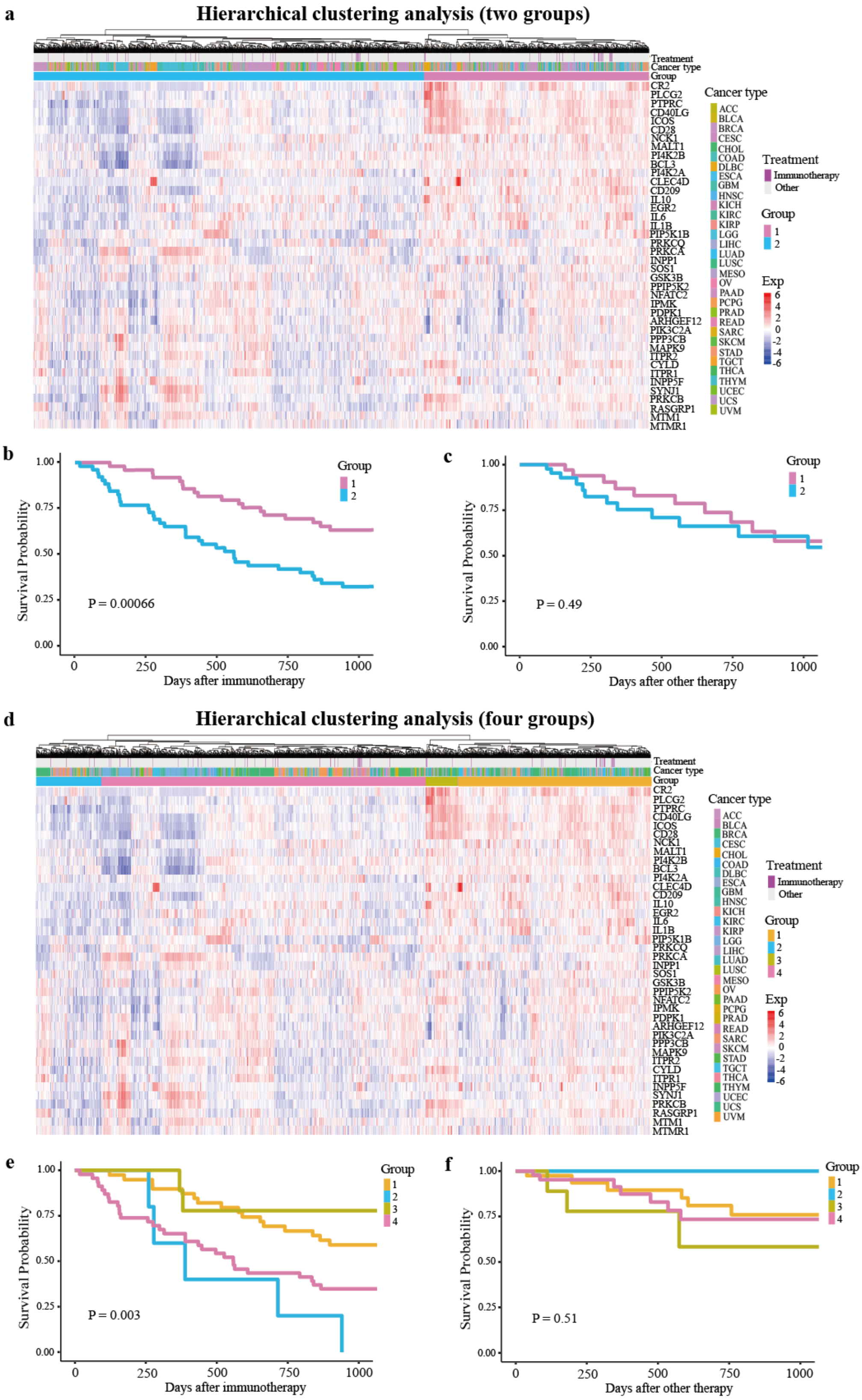

Supplementary Figure 4. Hierarchical clustering analysis based on the expression of the top 40

IPM genes.

a 2,836 patients across 32 cancer types were clustered into two groups. **b** The survival of patients receiving immunotherapy differed significantly in the groups. **c** No significant survival difference was observed among the groups of patients receiving non-immunotherapy. **d** 2,836 patients across 32 cancer types were clustered into four groups. **e** The survival of patients receiving immunotherapy differed significantly in the groups. **f** No significant survival difference was observed among the groups of patients receiving non-immunotherapy.

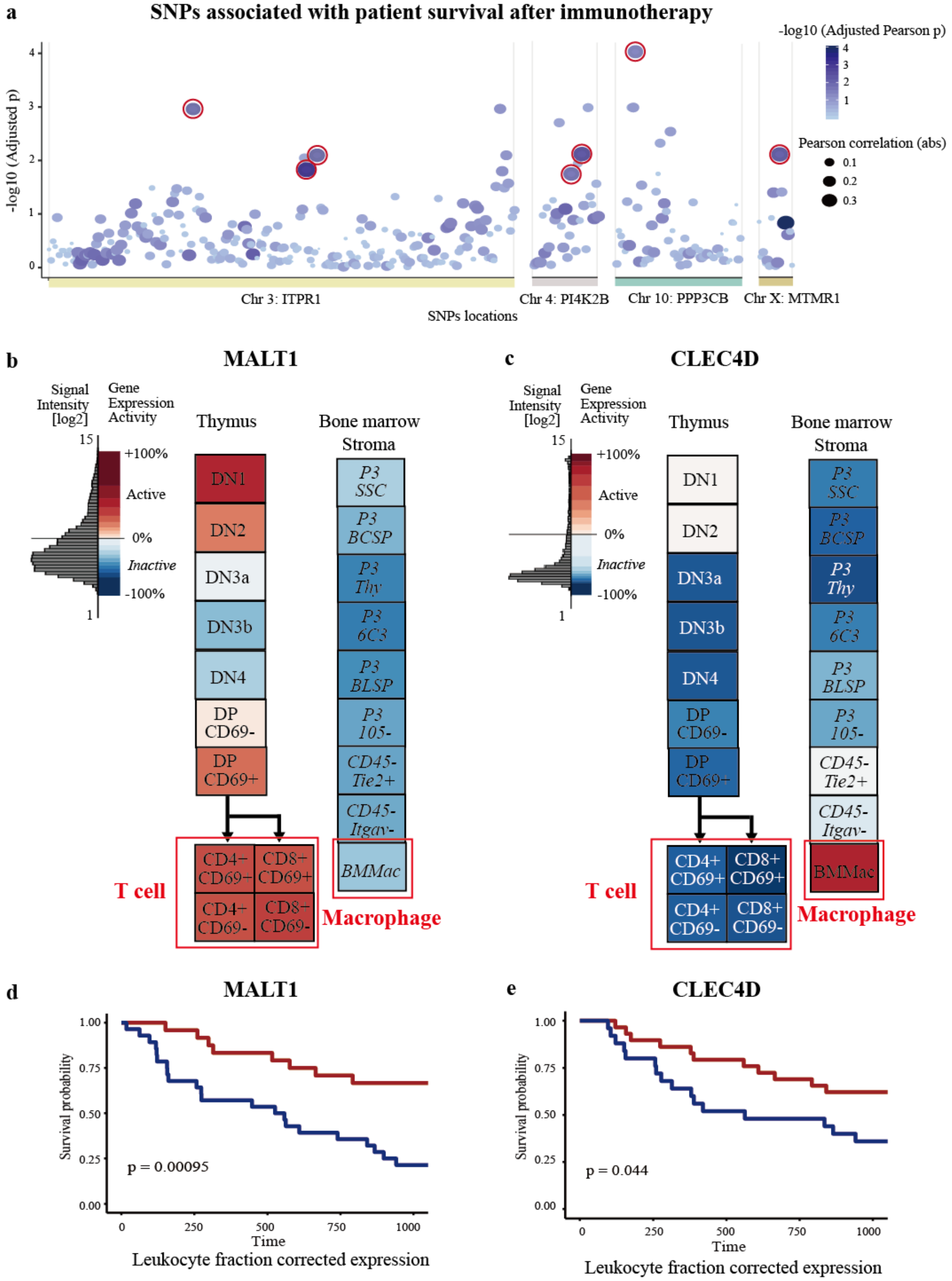

Supplementary Figure 5. Associations of gene expression with patient survival.

a SNPs associated with both the expression of IPMGs and the patient survival after immunotherapy. The Y axis is the -$\log_{10}$ p-values of GWAS adjusted by 1,000 permutations. The X axis is the relative genomic positions of the SNPs. Size of each points indicates the absolute value of the Pearson correlation coefficient between the expression of the IPMGs and the genotype of the SNPs within the gene region. Seven SNPs meeting statistical significance are circled in red. **b** Malt1 is primarily expressed in T cells based on mouse microarray data (https://gexc.riken.jp/models/1649/genes/Malt1?q=Malt1). **c** Clec4d is primarily expressed in macrophages based on mouse microarray data (https://gexc.riken.jp/models/1649/genes/Clec4d). **d, e** The expression level of MALT1 (CLEC4D) was positively associated with the improved survival of immunotherapy-treated patients based on fraction corrected expression.

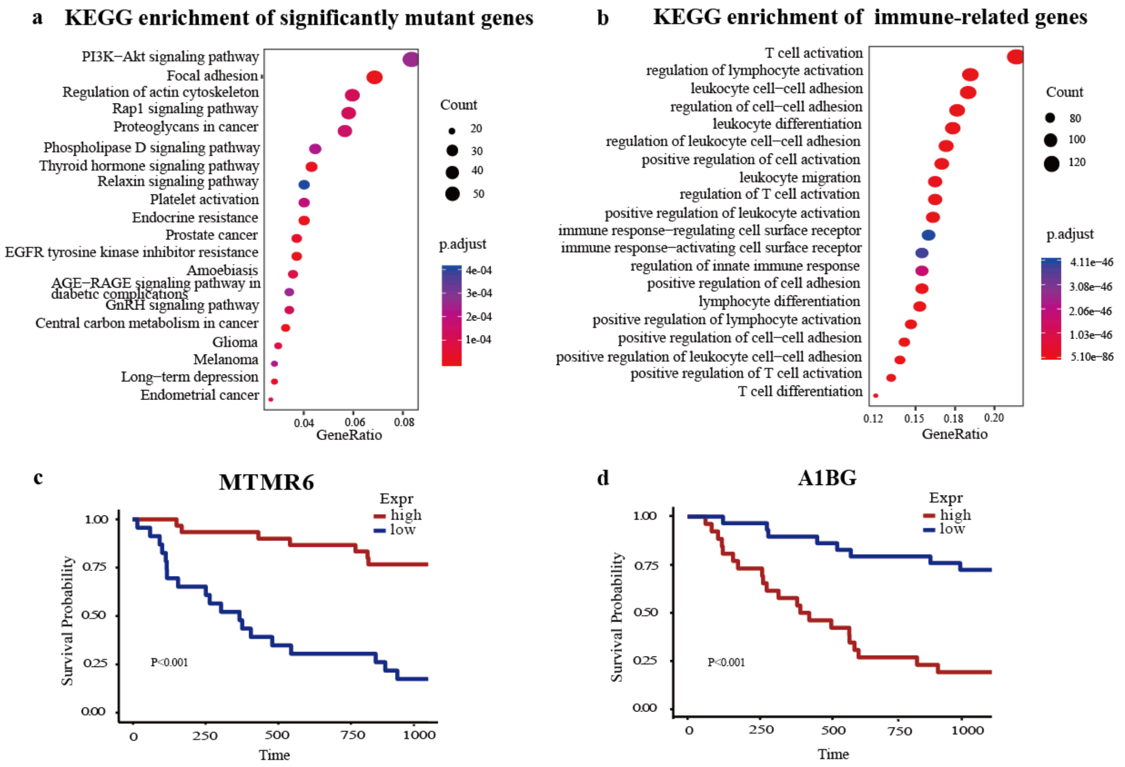

Supplementary Figure 6. The results of KEGG enrichment and survival analysis.

a KEGG enrichment of 1,686 significantly mutant genes demonstrates that most are related to oncogenic signaling pathways and development of cancers. **b** 676 genes were identified as those with an expression level that showed a positive correlation with leukocyte fraction but negative correlation with tumor proportion across all tumor samples. Go enrichment of these genes demonstrates that most are related to immune processes. **c, d** Kaplan-Meier curves of patients stratified by gene expression. MTMR6 is one of genes of which higher expression was associated with better survival of patients (c). A1BG is one of genes of which higher expression was associated with lower survival of patients (d).

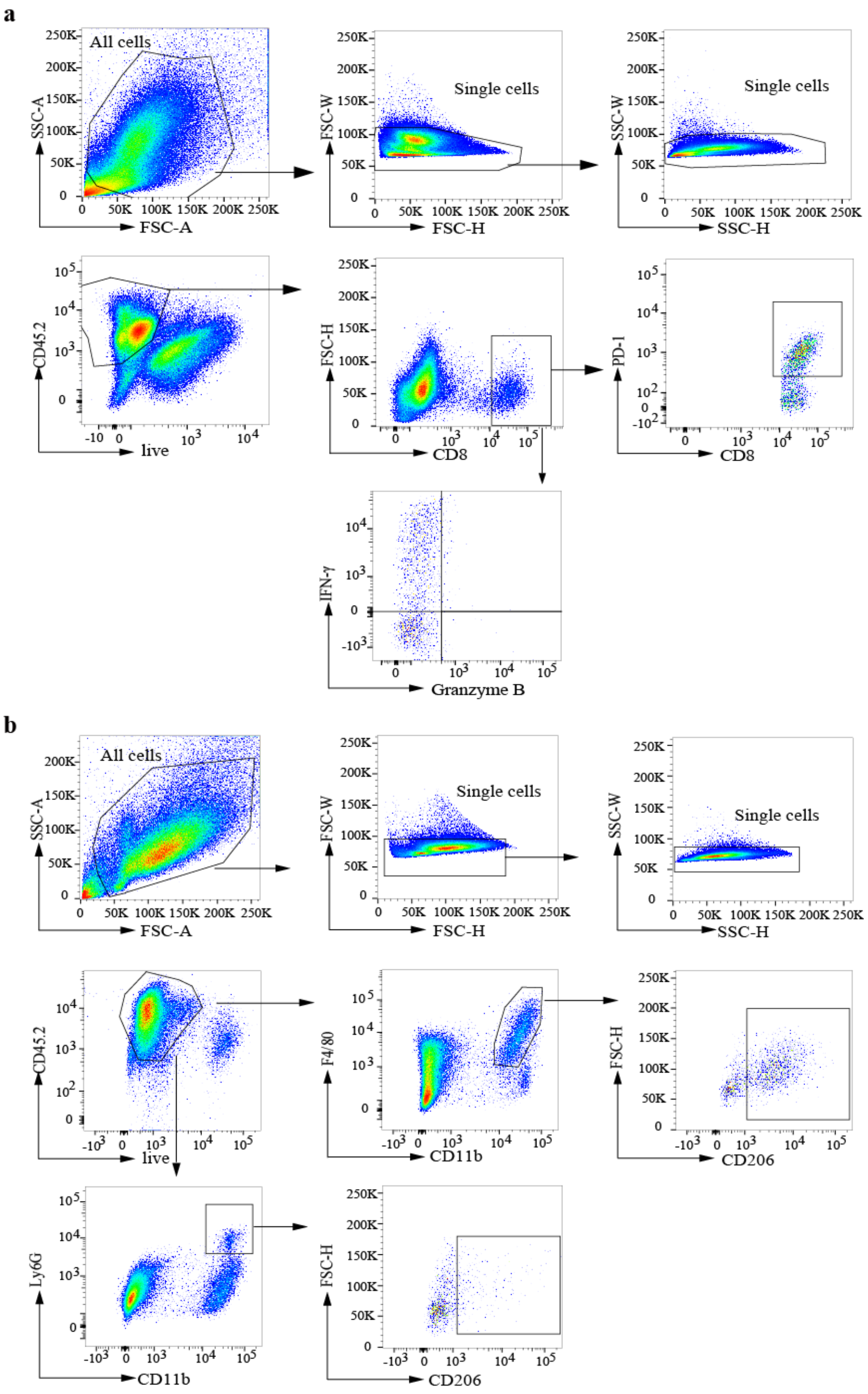

Supplementary Figure 7. Gating strategies used in FACS analysis.

a Single cells were prepared by using collagenase digestion for FACS analysis. Immune cells were firstly gated by FSC-A and SSC-A to exclude the debris, followed by FSC-H and FSC-W, SSC-H and SSC-W to gate the single cells. Dead cells were excluded by using viability dye, and the second gate is based on CD45. CD8+ cells were then gated by CD8+. All populations were further characterized by expression of PD-1, IFN-γ and GZMB. **b** Myeloid cells were defined by following gating strategies: CD11b+Ly6G+ to identify MDSC, CD11b+F4/80+ to identify macrophages. Each subset was analyzed for expression of CD206.

Un-cropped Images of Blots

Figure 5a

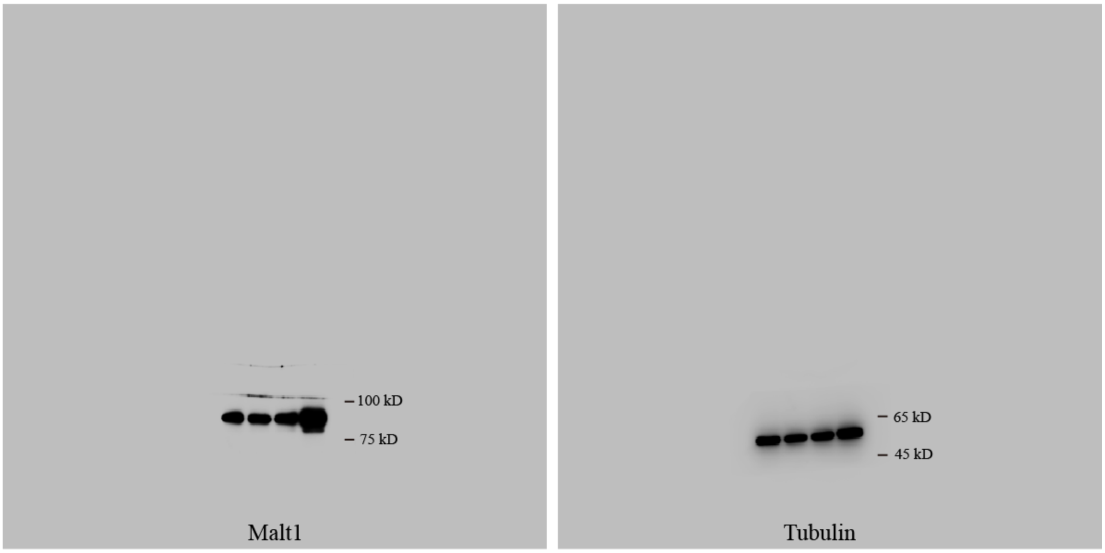

Supplementary Data

Supplementary Data 1 - Clinical data of TCGA patients with complete information and 3-years survival data of patients with matched expression data.

Supplementary Data 2 - A list of pathways associated with patient survival after immunotherapy.

Supplementary Data 3 - A list of feature importance scores of selected pathways using RSF model.

Supplementary Data 4 - A list of IPMGs positively associated with the improved prognosis of patients after immunotherapy.

Supplementary Data 5 - A list of active pathways enriched in groups of patients with high or low survival rate.

Supplementary Data 6 - A list of SNPs associated with patient survival after immunotherapy.
