# Supplementary material for: Knockout of immunotherapy prognostic marker genes eliminates the effect of the anti-PD-1 treatment
Source: NPJ Precis Oncol. 2021 May 7;5:37. doi: 10.1038/s41698-021-00175-2 (PMC8105367; doi:10.1038/s41698-021-00175-2)
Supplement: Supplementary file 8 — Reporting Summary [file 41698_2021_175_MOESM8_ESM.pdf]

nature research

Corresponding author(s): DBPR NPJPRECISIONONCOLOGY-00853R

Last updated by author(s): Dec 25, 2020

Reporting Summary

Nature Research wishes to improve the reproducibility of the work that we publish. This form provides structure for consistency and transparency in reporting. For further information on Nature Research policies, see our Editorial Policies and the Editorial Policy Checklist.

Statistics

For all statistical analyses, confirm that the following items are present in the figure legend, table legend, main text, or Methods section.

n/a	Confirmed	
☐	☒	The exact sample size (n) for each experimental group/condition, given as a discrete number and unit of measurement
☐	☒	A statement on whether measurements were taken from distinct samples or whether the same sample was measured repeatedly
☐	☒	The statistical test(s) used AND whether they are one- or two-sided<br>*Only common tests should be described solely by name; describe more complex techniques in the Methods section.*
☒	☐	A description of all covariates tested
☐	☒	A description of any assumptions or corrections, such as tests of normality and adjustment for multiple comparisons
☐	☒	A full description of the statistical parameters including central tendency (e.g. means) or other basic estimates (e.g. regression coefficient) AND variation (e.g. standard deviation) or associated estimates of uncertainty (e.g. confidence intervals)
☐	☒	For null hypothesis testing, the test statistic (e.g. F, t, r) with confidence intervals, effect sizes, degrees of freedom and P value noted<br>*Give P values as exact values whenever suitable.*
☒	☐	For Bayesian analysis, information on the choice of priors and Markov chain Monte Carlo settings
☒	☐	For hierarchical and complex designs, identification of the appropriate level for tests and full reporting of outcomes
☐	☒	Estimates of effect sizes (e.g. Cohen's d, Pearson's r), indicating how they were calculated

Our web collection on statistics for biologists contains articles on many of the points above.

Software and code

Policy information about availability of computer code

Data collection	No software was used in this study for data collection, just open-accessed web tools were used.
Data analysis	Publicly available software: R version 3.5.1 (https://www.R-project.org/). All R packages used are described in the Methods section.

For manuscripts utilizing custom algorithms or software that are central to the research but not yet described in published literature, software must be made available to editors and reviewers. We strongly encourage code deposition in a community repository (e.g. GitHub). See the Nature Research guidelines for submitting code & software for further information.

Data

Policy information about availability of data

All manuscripts must include a data availability statement. This statement should provide the following information, where applicable:

- Accession codes, unique identifiers, or web links for publicly available datasets
- A list of figures that have associated raw data
- A description of any restrictions on data availability

RNA-seq data are available from the NCBI GEO Database under accession number GSE158056. Other public data used in the current study are available in the TCGA (http://cancergenome.nih.gov).

Field-specific reporting

Please select the one below that is the best fit for your research. If you are not sure, read the appropriate sections before making your selection.

☒ Life sciences ☐ Behavioural & social sciences ☐ Ecological, evolutionary & environmental sciences

For a reference copy of the document with all sections, see nature.com/documents/nr-reporting-summary-flat.pdf

Life sciences study design

All studies must disclose on these points even when the disclosure is negative.

Sample size	All tumor samples available in open-accessed databases were chosen for analyses in this study. We analyzed RNA-seq data of 19 mice tumor tissues (WT mice received IgG: n=6; WT mice received anti-PD-1: n=6; KO mice received IgG: n=3; KO mice received anti-PD-1: n=4).
Data exclusions	No data were excluded.
Replication	The experiments in mouse models were done in triplicate or more and repeated twice or more, with similar observations.
Randomization	Mice were allocated to experimental groups based on their genotype and randomized within the given sex- and age- matched group.
Blinding	For mouse studies, mice were randomly assigned to treatment group prior to therapeutic administration and analysis of tumour weight, infiltration and cytokine productions.

Reporting for specific materials, systems and methods

We require information from authors about some types of materials, experimental systems and methods used in many studies. Here, indicate whether each material, system or method listed is relevant to your study. If you are not sure if a list item applies to your research, read the appropriate section before selecting a response.

Materials & experimental systems

n/a	Involved in the study	
☐	☒	Antibodies
☐	☒	Eukaryotic cell lines
☒	☐	Palaeontology and archaeology
☐	☒	Animals and other organisms
☒	☐	Human research participants
☒	☐	Clinical data
☒	☐	Dual use research of concern

Methods

n/a	Involved in the study	
☒	☐	ChIP-seq
☐	☒	Flow cytometry
☒	☐	MRI-based neuroimaging

Antibodies

Antibodies used	Fixable Viability Dye eFluor 506, APC-eFluor780 anti-CD45.2 (clone 104), FITC anti-CD45 (clone 30-F11), PerCP-Cyanine5.5 anti-CD45.2 (clone 104), PE anti-CD8 (clone 53-6.7), APC-eFluor 780 anti-CD8 (clone 53-6.7), eFluor 450 anti CD279(PD-1) (clone J43), APC anti-IFN-γ (clone XMG1.2), eFluor 450 anti-Granzyme B (clone NGZB), PerCP-Cyanine5.5 anti-CD11b (clone M1/70), PE anti-F4/80 (clone BM8), BV421 anti-CD206 (clone C068C2), Anti-PD-1 (J43).
Validation	All antibodies used in this work were purchased from companies, and validated by the manufacturers and by extensive use in published work.<br>-Fixable Viability Dye eFluor 506; Cat#65-0866-18<br>-APC-eFluor780 anti-CD45.2 (clone 104); Cat#47-0454080; RRID: AB_1272211<br>-FITC anti-CD45 (clone 30-F11);Cat#11-0451-82; RRID: AB_465050<br>-PerCP-Cyanine5.5 anti-CD45.2 (clone 104); Cat#45-0454-82; RRID: AB_953590<br>-PE anti-CD8 (clone 53-6.7); Cat#MA1-10304; RRID: AB_11156191<br>-APC-eFluor 780 anti-CD8 (clone 53-6.7); Cat#47-0081-82; RRID: AB_1272185<br>-eFluor 450 anti CD279(PD-1) (clone J43); Cat#48-9985-82; RRID: AB_2574139<br>-APC anti-IFN-γ (clone XMG1.2); Cat#505810; RRID: AB_315404<br>-eFluor 450 anti-Granzyme B (clone NGZB); Cat#48-8898-82; RRID: AB_11149362<br>-PerCP-Cyanine5.5 anti-CD11b (clone M1/70); Cat#45-0112-82; RRID: AB_953558<br>-PE anti-F4/80 (clone BM8); Cat#12-4801-82; RRID: AB_465923<br>-BV421 anti-CD206 (clone C068C2); Cat#141717; RRID: AB_2562232<br>-Anti-PD-1 (J43); Cat#BE0033; RRID: AB_1107747

Eukaryotic cell lines

Policy information about cell lines

Cell line source(s)	E.G7-OVA tumor cells were kindly provided by Dr. Chen Dong Lab (Tsinghua University, Beijing, China).
Authentication	The cell line was used authorized.
Mycoplasma contamination	All cells were confirmed mycoplasma negative at time of use.
Commonly misidentified lines (See ICLAC register)	N/A

Animals and other organisms

Policy information about studies involving animals; ARRIVE guidelines recommended for reporting animal research

Laboratory animals	Mouse B6. Malt1-/-, sourced from Ruefli-Brasse et al., 2003.<br>Mouse B6. Malt1fl/fl, sourced from Cheng et al., 2019.<br>Mouse B6. Dectin3-/-, sourced from Zhu et al. 2013.
Wild animals	N/A
Field-collected samples	N/A
Ethics oversight	The mouse experiments were conducted following the institutional guidelines and were approved by the Institutional Animal Care and Use Committees at Tsinghua University.

Note that full information on the approval of the study protocol must also be provided in the manuscript.

Flow Cytometry

Plots

Confirm that:

☒ The axis labels state the marker and fluorochrome used (e.g. CD4-FITC).

☒ The axis scales are clearly visible. Include numbers along axes only for bottom left plot of group (a 'group' is an analysis of identical markers).

☒ All plots are contour plots with outliers or pseudocolor plots.

☒ A numerical value for number of cells or percentage (with statistics) is provided.

Methodology

Sample preparation	For analyzing tumor-infiltrating cells in mouse model, tumors were digested with 1mg/ml Type 2 collagenase (Worthington) in the presence of 10U/ml DNase I for 1h at 37? prior to centrifuge on a 40% and 70% discontinuous Percoll gradient (GE Healthcare). The isolated cells were incubated with antibodies as follows: APC-eFluor 780 α-CD8 (clone 53-6.7), eFluor 450 α-PD-1 (clone RMPI-30), PE α-Granzyme B (clone NGZB) were purchased from eBioscience; APC α-IFN-γ (clone XMG1.2) was purchased from Biolegend; FITC α-Ly6G (clone 1A8), Alexa Flour700 α-Ly6C (clone AL-21) were purchased from BD Biosciences. For cytokine staining, cells were stimulated with 50ng/ml PMA (Sigma) and 500ng/ml Ionomycin (Sigma) in the presence of GolgiStop (BD Biosciences) for 5h at 37? and stained for cell surface markers followed by fixation/ permeabilization and intracellular cytokine staining (BD Biosciences).
Instrument	LSR Fortessa cytometers (BD Biosciences)
Software	FlowJo software
Cell population abundance	Cell yields from tumors are reported in the manuscript.
Gating strategy	Single cells were prepared by using collagenase digestion for FACS analysis. Immune cells were firstly gated by FSC-A and SSC-A to exclude the debris, followed by FSC-H and FSC-W, SSC-H and SSC-W to gate the single cells. Dead cells were excluded by using viability dye, and the second gate is based on CD45. Populations were then gated on specific antibody staining i.e CD8+ to identify CD8+ cells, CD11b+Ly6G+ to identify MDSC, CD11b+F4/80+ to identify macrophage.

☒ Tick this box to confirm that a figure exemplifying the gating strategy is provided in the Supplementary Information.
